# Assessment of the Potential Energy Hypersurfaces in Thymine within Multiconfigurational Theory: CASSCF vs. CASPT2

**DOI:** 10.3390/molecules21121666

**Published:** 2016-12-03

**Authors:** Javier Segarra-Martí, Antonio Francés-Monerris, Daniel Roca-Sanjuán, Manuela Merchán

**Affiliations:** 1 Instituto de Ciencia Molecular, Universitat de València, P. O. Box 22085, ES-46071 Valencia, Spain; Antonio.Frances@uv.es (A.F.-M.); Manuela.Merchan@uv.es (M.M.); 2 Present Address: Laboratoire de Chimie UMR 5182, École Normale Supérieure de Lyon, CNRS, Université de Lyon, 46 Allée d’Italie, F-69364 Lyon Cedex 07, France

**Keywords:** CASSCF/CASPT2, photochemistry, DNA, thymine, photostability

## Abstract

The present study provides new insights into the topography of the potential energy hypersurfaces (PEHs) of the thymine nucleobase in order to rationalize its main ultrafast photochemical decay paths by employing two methodologies based on the complete active space self-consistent field (CASSCF) and the complete active space second-order perturbation theory (CASPT2) methods: (i) CASSCF optimized structures and energies corrected with the CASPT2 method at the CASSCF geometries and (ii) CASPT2 optimized geometries and energies. A direct comparison between these strategies is drawn, yielding qualitatively similar results within a static framework. A number of analyses are performed to assess the accuracy of these different computational strategies under study based on a variety of numerical thresholds and optimization methods. Several basis sets and active spaces have also been calibrated to understand to what extent they can influence the resulting geometries and subsequent interpretation of the photochemical decay channels. The study shows small discrepancies between CASSCF and CASPT2 PEHs, displaying a shallow planar or twisted ^1^(ππ*) minimum, respectively, and thus featuring a qualitatively similar scenario for supporting the ultrafast bi-exponential deactivation registered in thymine upon UV-light exposure. A deeper knowledge of the PEHs at different levels of theory provides useful insight into its correct characterization and subsequent interpretation of the experimental observations. The discrepancies displayed by the different methods studied here are then discussed and framed within their potential consequences in on-the-fly non-adiabatic molecular dynamics simulations, where qualitatively diverse outcomes are expected.

## 1. Introduction

Over the last decade, a great deal of work has been done and yet plenty of controversy still surrounds the characterization of the main intrinsic photochemical mechanisms governing the photoinduced processes occurring in the DNA nucleobases [1,2,3,4,5,6,7,8,9,10,11]. Insight into their photochemical pathways provides unique information to ascertain the effect of light irradiation on the cellular system and is tightly related to the ability possessed by the genetic material to dissipate the excess of energy gained upon absorption in a harmless manner. This feature, known as photostability [12,13], is ascribed to the ultrafast decay of the excited state population to the ground state in a non-radiative manner mediated by conical intersections (CIs) between the initially accessed bright ^1^(ππ*) and the ground state, justifying the sub-ps deactivation registered experimentally [3] and explaining the reduced damage registered in DNA despite our constant UV-light exposure [14,15].

In order to understand the main photochemical decay routes occurring in DNA irradiated with UV light, countless theoretical and experimental efforts have been made yielding a solid background to the process, yet plenty of controversy remains [2,7,16,17,18,19]. Both static [20,21,22,23,24,25,26,27,28,29,30,31,32,33,34] and dynamic [16,17,35,36,37,38,39,40,41] theoretical studies have been performed over the last decade, the former usually employing more accurate and correlated methods due to their lower computational cost. Multiconfigurational methods are usually the preferred choice, particularly the complete active space self-consistent field (CASSCF) [42] and its second-order perturbation theory extension (CASPT2) [43]. These methods offer an even-footing treatment for all ground and excited-states partaking in the photo-process and are thus expected to properly represent the intricate PEHs and their minima/crossings [44,45,46,47,48]. These not only provide the PEHs for mechanistic purposes, but can also yield excited state absorptions [49,50], cationic energies [41], and other related observables with direct experimental counterparts [51,52]. Experimentally, several techniques have been employed to study the photoinduced phenomena of DNA nucleobases, ranging from pump-probe [4,28,53,54,55], time-resolved infrared [56,57,58,59], photoelectron [17,41,60,61] and recently even Auger spectroscopy [16]. This has allowed postulating different theoretical models to explain the photochemical decay paths of the canonical nucleobases and simulate a range of experimental spectroscopic observables, providing a molecular counterpart. However, the models accounting for the different experimental signals can strongly deviate from one another and be based on quite diverse potential energy hypersurfaces (PEHs), displaying discrepancies as to which are actually the main decay channels followed upon absorption or which states are the main protagonists in the photo-reaction [8].

**Figure 1:**
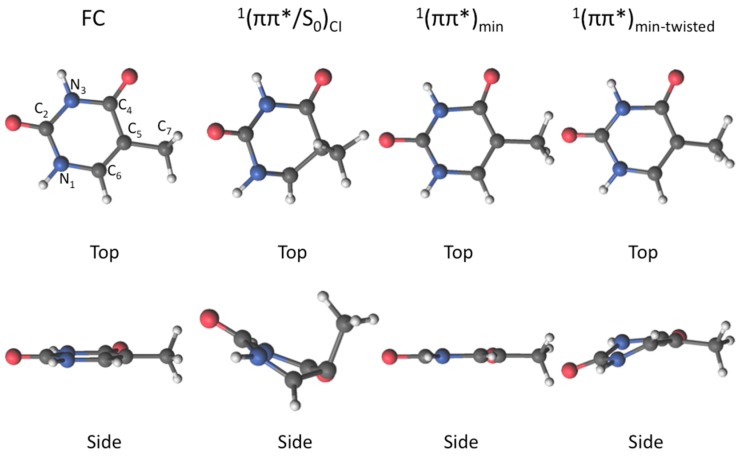
Geometry and labeling for the Franck-Condon region and the different structures found in thymine along its ^1^(ππ*) lowest-lying bright excited state potential energy hypersurface.

Several components in different time ranges have been detected over the years in time resolved experiments on DNA nucleobases: in the sub-ps regime, a bi-exponential decay was characterized for nucleobases, nucleosides or nucleotides [3,24,60,62], being mainly ascribed to ultrafast reactions along the initially accessed ^1^(ππ*) PEH, whereas a single component has also been observed in the few-ps timescale and being ascribed to dark nπ* states [55], and a longer component closer to the ns regime has been attributed to the eventual involvement of triplet states [55,56]. Theoretically, most studies agree in the initial involvement of the ^1^(ππ*) state for the sub-ps channels, having characterized a series of key structures in its PEH that rationalize its concrete role. Figure 1 displays these structures for thymine, the system under study in the present work. They correspond to two different excited state minima, one planar [11] and one partially twisted or “boat-like” found in more correlated computations or solvated environments [24,34,63,64], and an “ethene-like” CI [65,66] connecting the ^1^(ππ*) and ground states. The CI is usually employed to rationalize the ultrafast decay of thymine [8] and the extremely low fluorescence quantum yields recorded experimentally [62], and was initially reported in the pioneering study of Perun et al.[22]. The involvement of either dark nπ* or triplet states are usually ascribed to non-adiabatic interactions along the deactivation routes in these systems, which populate nπ* states that can in turn funnel the population towards the triplet manifold via intersystem crossing [67] given the large spin-orbit couplings featured [30,68,69], in agreement with El-Sayed rules [70].

In this paper we focus on the assessment of different multiconfigurational electronic structure theory approaches and geometry optimization techniques to benchmark the PEHs of the lowest-lying excited states in thymine and calibrate the extent to which the theoretical method employed can influence the model put forth to explain the photoinduced phenomena in DNA. To this end, widely used CASSCF approaches are compared to more expensive but currently feasible dynamically correlated CASPT2 methods, elucidating whether more correlated approaches are essential to achieve a proper description of the photoinduced phenomena. This comparison on static computations draws out the importance of the electron dynamic correlation to describe the PEHs and subsequent spectroscopic magnitudes for understanding the photoinduced processes occurring in thymine, and by extension other DNA or RNA nucleobases.

## 2. Results

The results are presented in three sections. First, calculations making use of the CASPT2//CASSCF protocol are discussed, focusing on the most important and initially accessed ^1^(ππ*) state. In this protocol, the geometries are optimized with the CASSCF method and the energies are computed with the CASPT2 method at the optimized structures. Next, results from CASPT2//CASPT2 computations are given. In this case, the geometries are also determined with the highest level of theory. A comparison between both methodologies is then drawn. The results obtained are summarized and discussed together with previous theoretical and experimental studies present in the literature, isolating the concrete role played by dynamic correlation in the PEH topology of the different photo-activated states and establishing its influence on our subsequent interpretation of the experimental evidence.

### 2.1. CASPT2//CASSCF

Minimum energy paths (MEPs) and unconstrained geometry optimizations are employed to map the PEH of the ^1^(ππ*) state in thymine at the CASSCF level and for different one-electron basis sets (see Computational Details), being summarized in Table 1. As can be seen, different behaviors might be inferred depending on the electronic correlation added through the basis set and active space employed. The smallest basis set, 6-31G, yields a planar minimum in the PEH of the ^1^(ππ*) excited state in all cases, whereas the larger and more flexible 6-31G* gives rise to the ^1^(ππ*/S_0_)_CI_ crossing when either the optimization thresholds are enhanced or the MEP algorithm is employed.

**Figure 2:**
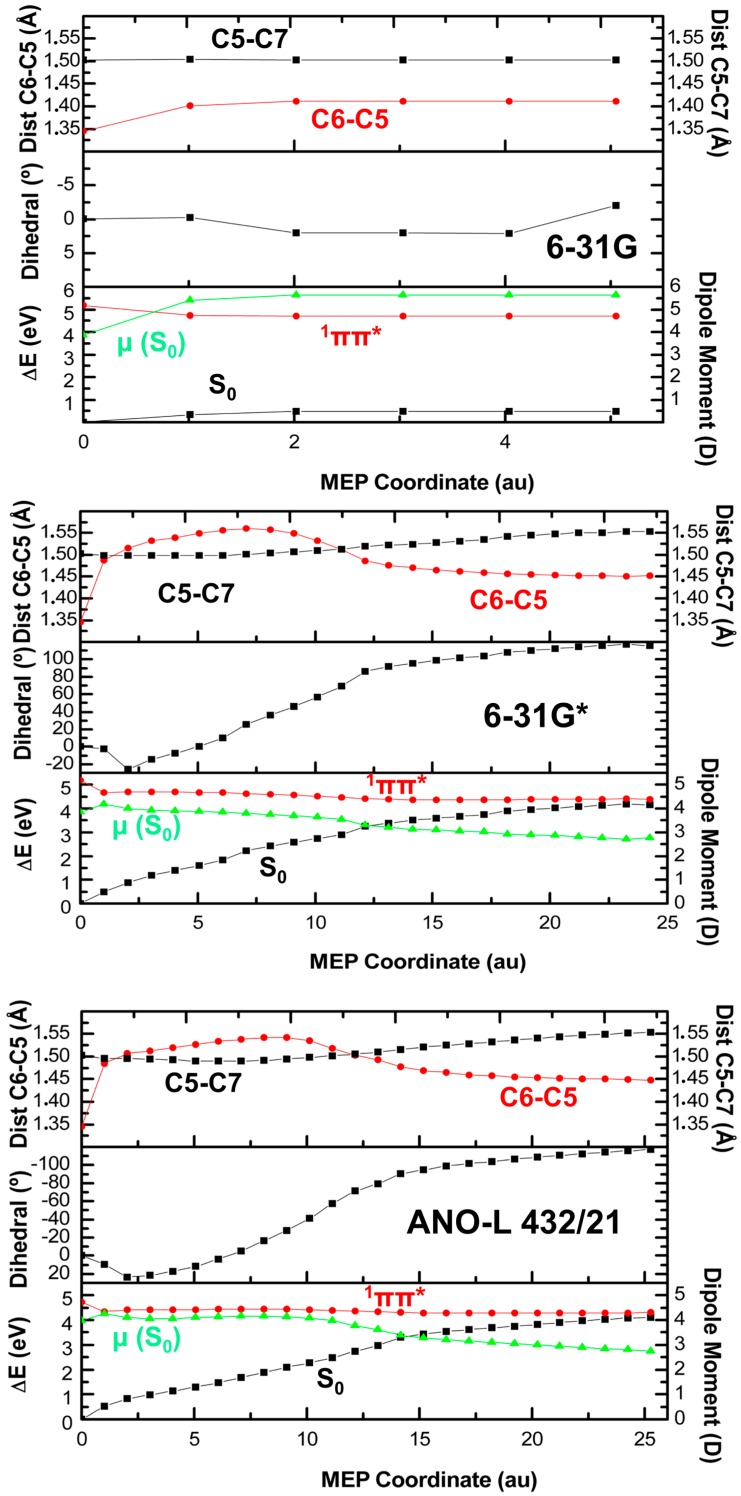
Complete active space second-order perturbation theory (CASPT2) energies and CASSCF dipole moments (μ) of the ground and lowest-lying ^1^(ππ*) excited state plus main geometrical distortions along the MEPs of the ^1^(ππ*) from the Franck-Condon region computed with the CASPT2//CASSCF(10,8) protocol and the 6-31G, 6-31G*, and atomic natural orbital (ANO)-L 432/21 basis sets. Results corresponding to the CASPT2//CASSCF(10,11)/6-31G*, CASPT2//CASSCF(10,8)/ANO-S 321/21, and CASPT2//CASSCF(10,8)/ANO-L 321/21 levels of theory can be found in Figure S1.

Figure 2 shows the computed MEPs by using different basis sets, increasing the amount of electron correlation considered. As can be seen, the MEP computed with the 6-31G basis set leads to the planar minimum ^1^(ππ*)_min_ in the ^1^(ππ*) hypersurface (see Figure 1), as previously reported by Perun et al. [22]. Using the same active space but increasing the flexibility of the one-electron basis set employed yields a very different result though (cf. Figure 2). In this case, the system decays in a barrierless manner towards the ring-puckering ethene-like crossing ^1^(ππ*/S_0_)_CI_. The same result holds true after adding extra correlating orbitals to the active space [CASSCF(10,11)] (see Figure S1), thus validating the previous result obtained with the 6-31G* basis set and the CASSCF(10,8) methodology. Active space selection and validation becomes essential as the size of the system under study increases and computations become impractical [64], and has also been documented to affect the outcome in photoinduced dynamics [71]. This illustrates the importance of the electronic correlation in order to give a proper description of the photochemical pathways actually accessible in a given molecular system [72]. Moreover, the technique employed to optimize the excited state seems to also play a role [8]. Geometry optimization of the ^1^(ππ*) state with the 6-31G* basis set yields the planar minimum ^1^(ππ*)_min_ (cf. Table 1), identical to the one found with the CASSCF/6-31G MEP, while the MEP at the CASSCF/6-31G* decays to ^1^(ππ*/S_0_)_CI_ as shown in Figure 2. In other words, for thymine, determination of the different stationary points independently (geometry optimization for the ground state and the excited state using standard convergence thresholds, and CI search) at the CASSCF/6-31G* level, and connecting those regions of the hypersurface subsequently by linear interpolation of internal coordinates (LIIC), offers a view of the photochemistry through a well-defined minimum on the excited state hypersurface. A similar behavior is observed when the geometry optimizations are carried out at the CASPT2 level, with the sole difference that the minimum found along the hypersurface can be slightly twisted or “boat-like” ^1^(ππ*)_min-twisted_ (cf. Figure 1) [17,34,63]. The conclusions derived from those studies seem to be dependent on the strategy used, highlighting the additional insights provided by more sophisticated MEP techniques to map PEHs, while stressing the need for computing experimental observables derived from accurate PEHs to be able to unambiguously compare face-to-face with the experiment [17,73].

To exert a more accurate treatment of the PEH, several ANO-type basis sets have been employed yielding qualitatively analogous results (see Figure 2 and Supplementary Materials), pointing to the direct decay towards the ground state as the main pathway to be followed upon light irradiation. Upon inspection of Table 1, it can be seen that, once extra polarization functions are added on the C,N,O atoms, as in 6-31G*, ANO-S 321/21, ANO-L 321/21, and ANO-L 432/21 basis sets, the resulting structures after geometry optimization/MEP with CASSCF(10,11) converge to the same ^1^(ππ*/S_0_)_CI_ structure with the exception of the CASSCF(10,11)/ANO-S 321/21 optimization, in which a planar minimum is obtained. A steady increase of electron correlation seems to converge towards the CI. The effect of the convergence thresholds on the PEHs has also been analyzed. While normal thresholds lead to minima in the rather flat ^1^(ππ*) PEH, the decrease of those values lead towards the CI, its resulting structures are in agreement with all other ^1^(ππ*/S_0_)_CI_ structures found and are described below.

Careful analysis shows a rather planar ^1^(ππ*) PEH that might explain the different results yielded by different thresholds and optimization procedures. Due to the planarity of the surface, and depending on the threshold employed during the optimization procedure, the presence of a given minimum may be warranted or not. This particularly flat topology of the PEH is not intrinsic to CASSCF/CASPT2, having been reported with other methods [24,73], and is in agreement with the broad emission band recorded and with its small fluorescence quantum yield [62].

The geometrical parameters defining the structures optimized at the different levels of theory are provided in the SI (Table S46). All results obtained employing basis sets with polarization functions are in agreement with those obtained by using the largest ANO-L 432/21. Deviations are small, as confirmed by the root mean square (RMS) averaged over all bond distances. In contrast, the smaller 6-31G basis set shows larger discrepancies. The C5-C6 and C5-C7 bond lengths and the dihedral describing the out of plane motion are the main geometrical distortions along the pyramidalization process towards the ring-puckering ^1^(ππ*/S_0_)_CI_, which strongly resembles those found for ethene [65]. The differences obtained for the geometry-optimized and MEP-resulting structures at the CASSCF(10,8) and CASSCF(10,11) levels are also shown in the SI (Table S47). Similar trends are found, supporting the fact that a steady increase of the correlation in the model leads directly towards the conical intersection.

In general, no qualitative changes on the topography of the PEH along the MEPs are discernible by going from the 6-31G* to ANO-L 432/21 (cf. Figure 2, Figures S1,S2), even though the vertical transition energies at the Franck-Condon region are overestimated on the former as compared to the results obtained with the ANO-type basis sets [49]. The computed values are 5.18, 4.83, 4.83, and 4.72 eV, with the 6-31G*, ANO-S 321/21, ANO-L 321/21, and ANO-L 432/21 basis sets, respectively, in agreement with the experimental band maximum placed at around 4.8 eV [3] and more correlated coupled cluster approaches [74]. Other properties highly dependent on the basis set, like the dipole moment, do change more prominently in the Pople-type basis set even though the mapped PEH appears to be qualitatively similar. This might have some implications in external perturbations to the wave function such as those given for QM/MM schemes [75,76,77], where solvation is described as dipole-driven electrostatic interactions between the solvent and the solute, and thus a more accurate characterization of the dipole moment may be important.

Additional computations have been carried out to explore the correlation effects in the less involved and dark ^1^(n_O_π*) state. CASSCF(14,10) has been employed in this case, adding both n_O_ lone-pair orbitals as described in the Computational Details to exert an even treatment to that given to the ^1^(ππ*) state, and only geometry optimizations have been carried out because its direct population upon absorption is discarded given its negligible oscillator strength. This active space has been shown to provide analogous results for the ^1^(ππ*) state as those previously depicted. A minimum on the ^1^(n_O_π*) PEH has been obtained, characterized by an elongated C4=O bond of ~1.37 Å for all basis set considered, and a C6C5C4O dihedral of −165.5° for the ANO-L 432/21 basis set, differing in 1–2 degrees depending on the basis set employed thus yielding qualitative akin results (see SI). Their overall RMS compared to ANO-L 432/21 is ~0.004, showing how covalent states such as ^1^(n_O_π*) show a lesser dependence on the dynamic correlation included in the model than that found for the ^1^(ππ*) state (cf. Table S48) [63]. It can therefore be concluded that 6-31G* provides a reasonable description of the PEH and will be the basis set used to test the adequacy of the CASPT2//CASSCF protocol against CASPT2//CASPT2.

### 2.2. CASPT2//CASPT2

Table 2 compiles the different results obtained by means of geometry optimizations/MEP calculations at the CASPT2//CASPT2 level of theory. As can be seen, the threshold in the optimization procedure plays an akin role to what has been shown at the CASSCF level, prompting towards ^1^(ππ*/S_0_)_CI_ when a tighter threshold is employed. The computed MEPs show a somewhat different trend compared to their CASSCF counterparts, in which the size of the hypersphere (r; see definition in Section 4) [78] employed along the path seems to influence the fate of the calculation. This appears to be inherent to the planarity of the ^1^(ππ*) state PEH as discussed above, and the hypersphere employed seems to be the deciding factor in this particular case, small r values being retained in the shallow minimum encountered along the flat PEH whereas larger r values skip the minimum and go straight to the CI. The results here strongly emphasize the planarity of the PEH from the immediate surroundings of the FC to the CI, being even flatter than at CASSCF, and agree with the low fluorescence quantum yield as well as the broad emission band registered [62], and with its ultrafast sub-ps deactivation reported experimentally [3,54,79].

The CASPT2 structures obtained in this section and their main parameters are analyzed in more detail in Table 3. As can be seen, the dihedral angle provides a good estimate to assess the resulting structure, as ^1^(ππ*)_min_^1^(ππ*)_twisted-min_, and ^1^(ππ*/S_0_)_CI_ feature values of ~0, 30, and 105°–110°, respectively, and the latter is similar to the ~120° angle obtained at CASSCF. Bond distances differ from CASSCF (cf. Table S47): C6-C5 bond distances are shorter for the planar minimum, while they appear to be slightly elongated for the CI geometry at the CASPT2 level. CASPT2 C5-C7 bond lengths, on the other hand, appear to be slightly shorter than the ~1.55 Å computed at CASSCF for the ^1^(ππ/S_0_)_CI_. Interestingly, MS-CASPT2 results seem to be closer to CASSCF values, and this supports the CASSCF description of the CI given the necessity to employ multistate treatments to recover the right dimensionality of the CI [80]. Regarding the new minimum identified in this section, ^1^(ππ*)_twisted-min_, C6-C5 and C5-C7 bond distances are ~1.45 and ~1.49 Å, respectively.

The CASPT2 method has additionally been benchmarked against MS-CASPT2 (see Table 2), a technique used previously to tackle the photochemical behavior of thymine [32,34,41]. The results here obtained are rather homogeneous, yielding similar estimates at both levels of theory. Regarding the MEP calculations, the MS-CASPT2 method appears to require slightly larger r values to reach the ^1^(ππ*/S_0_)_CI_, a result that does not appear in the geometry optimizations independently of the threshold employed. The ^1^(ππ*/S_0_)_CI_ structure obtained at the MS-CASPT2 level of theory resembles the one resulting from CASSCF calculations, whereas the CASPT2 CI is characterized by shorter bond length distances and a slightly lower dihedral angle. The differences obtained between CASPT2 and MS-CASPT2 relate to those previously reported for the penta-2-4-dieniminium cation (PSB-3) [81], where slight deviations are shown between these two different zeroth-order Hamiltonians. These could be partially caused by overestimated off-diagonal terms in the MS-CASPT2 effective Hamiltonian, as their definition can be lacking in some cases where the states included in the multistate procedure are of strongly different character, giving rise to an unphysical overestimation. Larger active spaces are demonstrated to palliate this effect but cannot be presently considered in this work to properly validate the MS-CASPT2 result, so we expect future results employing larger active spaces to be placed in between the estimates here given for the CASPT2 and MS-CASPT2 methods [81]. More accurate estimates are expected from the extended multistate (XMS) approach of Granovsky [82] and its related implementations [83,84,85,86,87,88].

**Figure 3:**
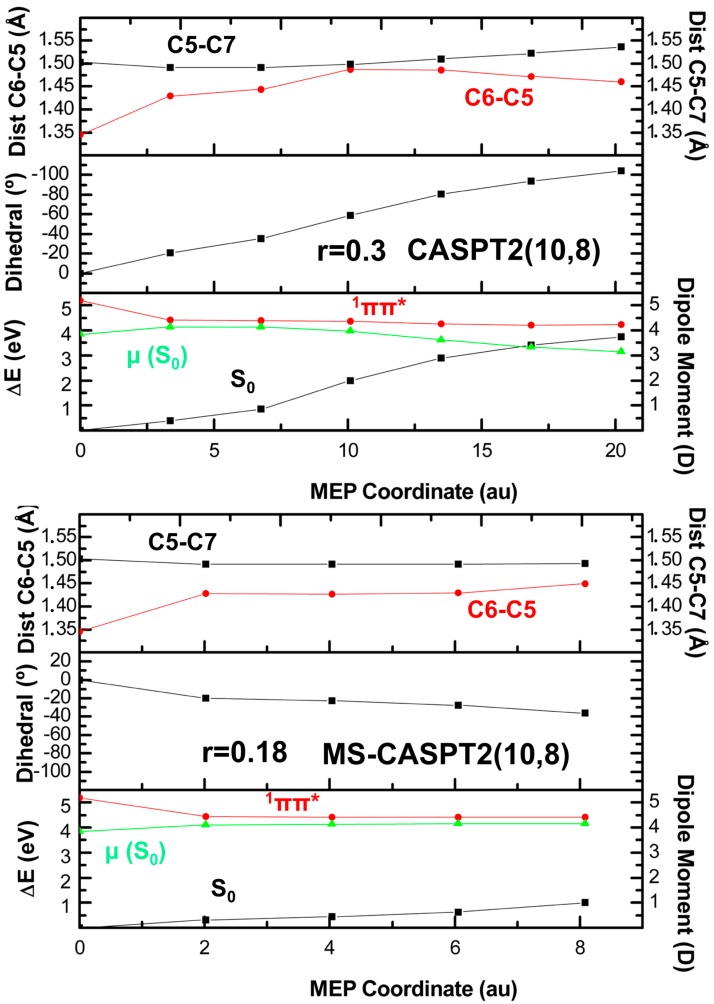
CASPT2 energies and CASSCF dipole moments (μ) of the ground and lowest-lying ^1^ππ* excited state plus main geometrical distortions along the MEPs of the ^1^ππ* from the Franck-Condon region computed with CASPT2//CASPT2 and MS-CASPT2//MS-CASPT2 with the 6-31G* basis set.

Representative MEPs yielding the different key structures on the ^1^(ππ*) state hypersurface are depicted in Figure 3. No planar structure is obtained by employing the MEP strategy at the CASPT2 level. Instead, the presence of a twisted minimum along the decay path, previously reported in the literature [34,63], appears to be the main structure hampering the barrierless route towards the ^1^(ππ*/S_0_)_CI_. This relates to what has also been found for PSB-3, where the dynamically correlated CASPT2 displays a more stable minimum under the planar minimum described by CASSCF [63]. Lower values of r favor this situation in which thymine finds itself to be stranded in a shallow minimum. Two different behaviors can be distinguished by inspection of Figure 3. In CASPT2/r = 0.09 (see Figure S3) and MS-CASPT2/r = 0.18 the MEPs lead towards a minimum, which is different from the planar minimum reported above at the CASSCF level. CASPT2/r = 0.30, on the other hand, exhibits a barrierless pathway towards the CI with the ground state. This finding is also obtained at the MS-CASPT2/r = 0.30 level with a slight difference in the dihedral angle and the C6-C5 and C5-C7 bond distances at the CI geometry.

As in CASPT2//CASSCF, additional computations have been carried out to characterize the ^1^(n_O_π*) minimum, employing CASSCF(14,10). The minimum has been computed at the CASPT2 and MS-CASPT2 levels employing the 6-31G* basis set. Shorter C4=O distances (~1.365 Å) are obtained as compared to those previously reported with the CASSCF method, and a nearly planar structure displaying a C6C5C4O dihedral of −178° (see SI), contrary to CASSCF findings. It is worth noting that both CASPT2 and MS-CASPT2 converge to the same structure, the choice of zeroth-order Hamiltonian exerting a lesser influence in this case as opposed to what has been previously reported for ^1^(ππ*), which can be related to the different nature of the states: zwitterionic (ππ*) and covalent (nπ*).

## 3. Discussion

CASSCF and CASPT2/MS-CASPT2 results obtained in the previous sections are here compared to assess the accuracy of the usually employed CASSCF method for geometry optimizations versus the CASPT2 method.

**Figure 4:**
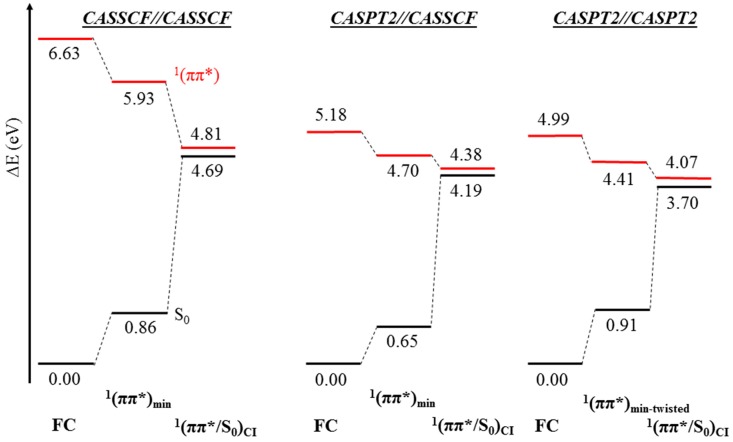
CASSCF//CASSCF, CASPT2//CASSCF, and CASPT2//CASPT2 energy diagrams of the key structures predicted to partake in the deactivation along the ^1^(ππ*) state. Results obtained from excited-state geometry optimizations and MEPs with the (10,8) active space and the 6-31G* basis set.

As can be seen in Figure 4, which summarizes the results displayed in Figure 2 and Figure 3 and Table 1 and Table 2, the results of the MEP [89] calculations at the CASPT2//CASSCF/6-31G* and CASPT2//CASPT2/6-31G* levels of theory exhibit in this particular case similar potential energy profiles that validate the CASPT2//CASSCF approach as an adequate protocol to predict the ultrafast nature of the decay process. However, it has been shown that the extremely flat ^1^(ππ*) PEH seems to have a certain dependence on the dynamic correlation in its description, featuring a different minimum and displaying distinct gradients along the PEH that are expected to yield more noticeable differences in non-adiabatic molecular dynamics simulations. In such simulations, either the CASSCF or the CASPT2 methods are used, but not a combination of both as in the static CASPT2//CASSCF protocol [89,90,91,92,93]. Whereas the mentioned small discrepancies are not expected to alter our vision of the photoinduced decay (ultrafast process) and the interpretation of the experimental lifetimes from a static standpoint, it is important to note that they may influence spectroscopic observables arising from the minima characterized, as magnitudes like excited state absorptions show a strong dependence on the molecular structure [94].

Some differences are encountered for the geometry of the ^1^(n_O_π*) minimum. Whereas CASSCF predicts a partially twisted structure, both CASPT2 and MS-CASPT2 yield completely planar geometries more similar to the ^1^(n_O_π*/ππ*)_CI_ crossing mediating the ^1^(n_O_π*) non-adiabatic population (see SI). The structural similarities featured between the ^1^(n_O_π*) minimum and ^1^(n_O_π*/ππ*)_CI_, as well as their energetic proximity, according to the CASPT2//CASSCF and CASPT2//CASPT2 protocols, may promote a re-crossing back to the ^1^(ππ*) manifold [25] that may explain the reduced ^1^(n_O_π*) yield registered experimentally [55]. This would drastically reduce the role of the ^1^(n_O_π*) state in the photo-process, in contrast to what has been reported in MRCIS and CASSCF non-diabatic molecular dynamics studies [26,93,95], where a vast fraction of the excited state population ends up trapped in the ^1^(n_O_π*) state in the sub-ps timescale. This incidence is related to the likely overestimated role of the ^1^(n_O_π*) PEH in MRCIS/CASSCF, where this state is proposed to be the main responsible of the ultrafast deactivation, and points towards an uneven treatment of the ^1^(n_O_π*) and ^1^(ππ*) states, which are described quite differently with methods lacking dynamic correlation like CASSCF [63,81], and that has motivated studies in the literature to assess the importance of the dynamic correlation in the overall photochemical picture [19,72].

**Figure 5:**
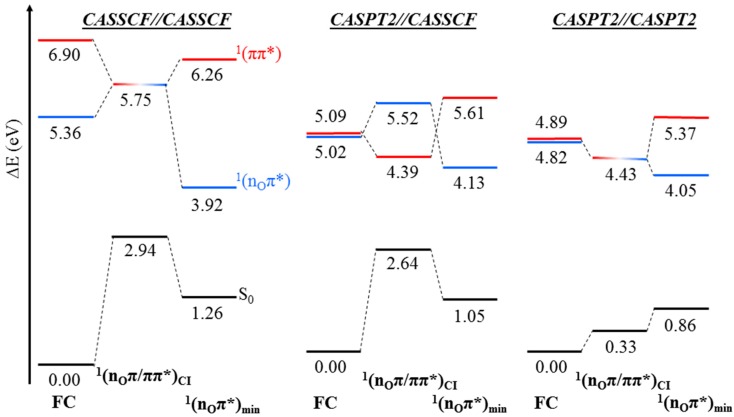
CASSCF//CASSCF, CASPT2//CASSCF, and CASPT2//CASPT2 energy diagrams of the key structures predicted to partake in the deactivation along the ^1^(n_O_π*) state. Results were obtained with the (14,10) active space and the 6-31G* basis set.

This is further assessed in Figure 5, where an energy diagram of the involvement of the ^1^(n_O_π*) state at both CASSCF//CASSCF and CASPT2//CASPT2 levels is shown, together with the energy profile arisen from the CASPT2//CASSCF protocol. Figure 5 depicts pronounced differences. The main ones are: *i*) in the Franck-Condon (FC) region, a large energy gap between the bright ^1^(ππ*) and dark ^1^(n_O_π*) states appears with the CASSCF//CASSCF approach, while at the CASPT2//CASPT2 level, the states appear to be near degenerate, and ii) the twisted CASSCF ^1^(n_O_π*) minimum appears to be much lower in energy with respect to ^1^(n_O_π*/ππ*)_CI_ than its planar CASPT2 counterpart. This artificial stabilization is corrected at the CASPT2//CASSCF level (cf. Figure 4). The former difference (*i*) will have important incidences in the early time events of molecular dynamics simulations employing the CASSCF method, where kinetic energy is built along the pathway from the initially accessed ^1^(ππ*) state towards the ^1^(n_O_π*/ππ*)_CI_ that may artificially increase the population on the ^1^(n_O_π*) state, whereas the latter discrepancy (*ii*) is expected to ensure the population trapping in the ^1^(n_O_π*) state. On the other hand, at the CASPT2//CASPT2 level, both ^1^(ππ*) and ^1^(n_O_π*) states are degenerate at the FC region and both ^1^(n_O_π*/ππ*)_CI_ and the ^1^(n_O_π*) minimum appear within half an eV, which is expected to increase the re-crossing back to the ^1^(ππ*) manifold [25] and thus reduce the population ending in the ^1^(n_O_π*) state [55].

## 4. Computational Details

CASSCF and CASPT2 methods have been used in this study as implemented in MOLCAS 7.6 [96,97]. Two different types of basis sets were used: i) the segmented 6-31G and 6-31G* basis sets, and ii) two generally contracted basis sets of atomic natural orbital (ANO) type with C,N,O(10s,6p,3d)/H(7s3p) [98] primitive functions contracted to C,N,O[3s2p1d]/H[2s1p] (hereafter ANO-S 321/21) and C,N,O(14s,9p,4d,3f)/H(8s4p3d) [99] primitive functions contracted to C,N,O[3s2p1d]/H[2s1p] and C,N,O[4s3p2d]/H[2s1p] (hereafter ANO-L 321/21 and ANO-L 432/21, respectively). These are selected to showcase the smallest basis set required for a correct characterization of the PEHs in thymine.

Multiconfigurational wave functions have been built using in the CAS space the whole valence π occupied and virtual orbitals of thymine, giving rise to the CASSCF(10,8). Further calculations with three extra π virtual orbitals have been performed to assess the convergence of the active space for such computations, resulting in the CASSCF(10,11) level [11]. No symmetry restrictions were imposed to the molecule as required to avoid any symmetry breaking problems and to allow complete freedom during the optimization procedures. Additional computations including the n_O_ lone pair orbitals in the active space were also carried out and labeled as CASSCF(14,10). Two roots were averaged for CASSCF(10,8) and CASSCF(10,11), whereas five states were included for CASSCF(14,10), accounting for both low-lying n_O_π* states. Second-order perturbative corrections have been computed on top of the CASSCF wave functions, maintaining all core electrons frozen during the perturbation step and making use of the zeroth-order Hamiltonian as originally implemented [43,48], and employing a 0.2 a.u. imaginary level shift to avoid weakly-interacting intruder states [100]. Additional computations at the multistate CASPT2 (MS-CASPT2) level have also been performed [101] for the sake of comparison with previous results present in the literature [34].

Geometry optimizations and minimum energy paths (MEPs) have been obtained at both CASSCF and CASPT2 levels. CASSCF MEPs have been computed with analytical gradients [102] and subsequent CASPT2 calculations have been performed on the converged geometries along the path in order to include dynamic correlation, as usually performed nowadays in photochemical studies following the CASPT2//CASSCF protocol [46]. CASPT2 optimizations and MEPs, on the other hand, have been computed, making use of numerical gradients. MEPs have been extensively used in this work, making use of mass weighted coordinates. This technique follows (if present) a steepest descent path, in which each step is built by the minimization of the energy on a hyperspherical cross section of the PEH of a predefined radius centered on the geometry optimized in the previous step, providing a powerful tool to study the photophysics and photochemistry of molecular systems [78]. Different optimization thresholds and hypersphere radii (r) for the MEPs have been computed to assess their influence in the PEH topography. For geometry optimizations, a standard convergence threshold of 10^−5^ and 10^−3^ (in a.u.) for the energy change and the norm of the gradient have been used, respectively. Additionally, a tighter threshold characterized by an energy change of 10^−8^ and a norm of the gradient of 10^−6^ has also been employed. On the other hand, several values for the hypersphere radius (r) defining the MEP have been computed, ranging from 0.09 to 0.3 (in a.u.). CIs are characterized at the CASSCF level with the GAUSSIAN 09 package [103] and employing the projection technique of Bearpark et al. [104], while CASPT2/MS-CASPT2 crossings are directly taken from the minimum energy crossing points found along the MEP.

## 5. Conclusions

We can conclude that the flat PEH of the ^1^(ππ*) bright low-lying excited state of thymine, widely discussed in the present study, presents a couple of shallow minima on the deactivation path towards the ^1^(ππ*/S_0_)_CI_, which might account for the sub-ps to few-ps signals registered experimentally. The different minima exhibit small barriers, within the intrinsic error of the methods employed, and thus the decay is predicted to be ultrafast, the fluorescence quantum yield is expected to be small, and the emissive feature broad, in accordance with the experimental evidence. The ^1^(n_O_π*) state, on the other hand, displays a slightly twisted minimum with the CASSCF method and a planar minimum with the CASPT2. In the latter case, the minimum of the ^1^(n_O_π*) state is placed at the vicinities of the ^1^(n_O_π*/ππ*)_CI_ crossing close to the Franck-Condon region which might facilitate the re-crossing towards the ^1^(ππ*) state and thus account for the reduced ^1^(n_O_π*) yield registered experimentally. Overall, CASPT2//CASSCF and CASPT2//CASPT2 (or MS-CASPT2//MS-CASPT2) appear to yield on static grounds a similar qualitative picture of the photoinduced phenomena in the sub-ps timescale. However, this is expected to change in molecular dynamics simulations where the overly steep CASSCF surfaces/energy gradients have been reported to predict too short decay times compared to those recorded. The study supports the qualitative picture obtained on static grounds of the CASPT2//CASSCF protocol, given the energetic discrepancies between CASSCF and CASPT2 are carefully assessed while calling for dynamically correlated CASPT2 gradients for an even-footing description of the photoinduced events on the different states involved in on-the-fly non-adiabatic molecular dynamics simulations. Novel spectroscopic techniques with enhanced temporal and spectral resolution should help overcome these ambiguities over the coming years [105,106,107].

## Figures and Tables

**Table 1:** Resulting structures for the complete active space self-consistent field (CASSCF) geometry optimizations and minimum energy paths (MEPs) computed on thymine.

Basis Set	Type of Calc.	**Active Space (e^−^ in Orbs)**	
		10in8	10in11
6-31G	Opt	^1^(ππ*)_min_	^1^(ππ*)_min_
	Opt Tight ^1^	^1^(ππ*)_min_	^1^(ππ*)_min_
	MEP	^1^(ππ*)_min_	^1^(ππ*)_min_
6-31G*	Opt	^1^(ππ*)_min_	^1^(ππ*/S_0_)_CI_
	Opt Tight ^1^	^1^(ππ*/S_0_)_CI_	^1^(ππ*/S_0_)_CI_
	MEP	^1^(ππ*/S_0_)_CI_	^1^(ππ*/S_0_)_CI_
ANO-S 321/21	Opt	^1^(ππ*)_min_	^1^(ππ*)_min_
	Opt Tight ^1^	^1^(ππ*/S_0_)_CI_	^1^(ππ*/S_0_)_CI_
	MEP	^1^(ππ*/S_0_)_CI_	^1^(ππ*/S_0_)_CI_
ANO-L 321/21	Opt	^1^(ππ*)_min_	^1^(ππ*/S_0_)_CI_
	Opt Tight ^1^	^1^(ππ*/S_0_)_CI_	^1^(ππ*/S_0_)_CI_
	MEP	^1^(ππ*/S_0_)_CI_	^1^(ππ*/S_0_)_CI_
ANO-L 432/21	Opt	^1^(ππ*)_min_	^1^(ππ*/S_0_)_CI_
	Opt Tight ^1^	^1^(ππ*/S_0_)_CI_	^1^(ππ*/S_0_)_CI_
	MEP	^1^(ππ*/S_0_)_CI_	^1^(ππ*/S_0_)_CI_

^1^ Convergence thresholds of 10^−8^ and 10^−6^ for the energy change and the norm of the gradient, respectively.

**Table 2:** CASPT2(10,8)/6-31G* geometry-optimization and MEP geometries obtained for thymine. r denotes the hypersphere radius employed in the calculation (in a.u.) [78].

Type of Calculation	Method	Structure
Opt	CASPT2	^1^(ππ*)_min_
	MS-CASPT2	^1^(ππ*)_min_
Opt Tight ^1^	CASPT2	^1^(ππ*/S_0_)_CI_
	MS-CASPT2	^1^(ππ*)_min-twisted_
MEP	CASPT2	
	r = 0.09	^1^(ππ*)_min-twisted_
	r = 0.18	^1^(ππ*/S_0_)_CI_
	r = 0.30	^1^(ππ*/S_0_)_CI_
	MS-CASPT2	
	r = 0.18	^1^(ππ*)_min-twisted_
	r = 0.30	^1^(ππ*/S_0_)_CI_

^1^ Convergence thresholds of 10^−8^ and 10^−6^ for the energy change and the norm of the gradient, respectively.

**Table 3:** Geometrical parameters describing the structures obtained with different geometry optimization and MEP procedures at the CASPT2(10,8)/6-31G* level of theory. Distances are given in Å and angles in degrees.

Type of Calc.	Method	Dihedral/HC6C5C7	Dist C6-C5	Dist C5-C7
Opt	CASPT2	0.0487	1.4331	1.4915
	MS-CASPT2	0.079	1.4302	1.4913
Opt Tight ^1^	CASPT2	98.2401	1.4666	1.5283
	MS-CASPT2	−30.063	1.4288	1.4902
MEP	CASPT2			
	r = 0.09	−33.8446	1.4505	1.4924
	r = 0.18	−102.8496	1.4625	1.5341
	r = 0.30	−103.7962	1.4607	1.5363
	MS-CASPT2			
	r = 0.18	−35.9473	1.4497	1.4924
	r = 0.30	−111.5802	1.4505	1.5405

^1^ Convergence thresholds of 10^−8^ and 10^−6^ for the energy change and the norm of the gradient, respectively.

## Supplementary Materials

The following are available online at www.mdpi.com/1420-3049/21/12/1666/s1: Cartesian coordinates of the minima and minimum energy crossing points obtained within the study, absolute energies of relevant structures, and additional MEP profiles are given.

## References

[1] Kleinermanns K., Nachtigallova D., de Vries M.S. (2013). Excited state dynamics of DNA bases. Int. Rev. Phys. Chem..

[2] Improta R., Santoro F., Blancafort L. (2016). Quantum mechanical studies on the photophysics and the photochemistry of nucleic acids and nucleobases. Chem. Rev..

[3] Crespo-Hernandez C.E., Cohen B., Hare P.M., Kohler B. (2004). Ultrafast excited-state dynamics in nucleic acids. Chem. Rev..

[4] Middleton C.T., de La Harpe K., Su C., Law Y.K., Crespo-Hernandez C.E., Kohler B. (2009). DNA excited-state dynamics: From single bases to the double helix. Annu. Rev. Phys. Chem..

[5] Schreier W.J., Gilch P., Zinth W. (2015). Early events of DNA photodamage. Annu. Rev. Phys. Chem..

[6] Gustavsson T., Improta R., Markovitsi D. (2010). DNA/RNA: Building blocks of life under UV irradiation. J. Phys. Chem. Lett..

[7] Barbatti M., Borin A., Ullrich S. (2014). Photoinduced processes in nucleic acids. Top. Curr. Chem..

[8] Giussani A., Segarra-Martí J., Roca-Sanjuán D., Merchán M. (2015). Excitation of nucleobases from a computational perspective I: Reaction paths. Top. Curr. Chem..

[9] Improta R., Barone V. (2015). Excited states behavior of nucleobases in solution: Insights from computational studies. Top. Curr. Chem..

[10] Serrano-Andrés L., Merchán M. (2009). Are the five natural DNA/RNA base monomers a good choice from natural selection? A photochemical perspective. J. Photochem. Photobiol. C-Photochem. Rev..

[11] Merchán M., González-Luque R., Climent T., Serrano-Andrés L., Rodriguez E., Reguero M., Pelaez D. (2006). Unified model for the ultrafast decay of pyrimidine nucleobases. J. Phys. Chem. B.

[12] Sobolewski A.L., Domcke W. (2006). The chemical physics of the photostability of life. Europhys. News.

[13] Serrano-Andrés L., Merchán M., Borin A.C. (2006). Adenine and 2-aminopurine: Paradigms of modern theoretical photochemistry. Proc. Natl. Acad. Sci. USA.

[14] Cadet J., Grand A., Douki T. (2015). Solar UV radiation-induced DNA bipyrimidine photoproducts: Formation and mechanistic insights. Top. Curr. Chem..

[15] Cadet J., Mouret S., Ravanat J.-L., Douki T. (2012). Photoinduced damage to cellular DNA: Direct and photosensitized reactions. Photochem. Photobiol..

[16] McFarland B.K., Farrell J.P., Miyabe S., Tarantelli F., Aguilar A., Berrah N., Bostedt C., Bozek J.D., Bucksbaum P.H., Castagna J.C. (2014). Ultrafast X-ray auger probing of photoexcited molecular dynamics. Nat. Commun..

[17] Hudock H.R., Levine B.G., Thompson A.L., Satzger H., Townsend D., Gador N., Ullrich S., Stolow A., Martinez T.J. (2007). Ab initio molecular dynamics and time-resolved photoelectron spectroscopy of electronically excited uracil and thymine. J. Phys. Chem. A.

[18] Shukla M.K., Leszczynski J. (2007). Electronic spectra, excited state structures and interactions of nucleic acid bases and base assemblies: A review. J. Biomol. Struct. Dyn..

[19] Barbatti M. (2014). Photorelaxation induced by water–chromophore electron transfer. J. Am. Chem. Soc..

[20] Merchán M., Serrano-Andrés L. (2003). Ultrafast internal conversion of excited cytosine via the lowest pp* electronic singlet state. J. Am. Chem. Soc..

[21] Sobolewski A.L., Domcke W. (2010). Molecular mechanisms of the photostability of life. Phys. Chem. Chem. Phys..

[22] Perun S., Sobolewski A.L., Domcke W. (2006). Conical intersections in thymine. J. Phys. Chem. A.

[23] Perun S., Sobolewski A.L., Domcke W. (2005). Ab initio studies on the radiationless decay mechanisms of the lowest excited singlet states of 9H-adenine. J. Am. Chem. Soc..

[24] Gustavsson T., Banyasz A., Lazzarotto E., Markovitsi D., Scalmani G., Frisch M.J., Barone V., Improta R. (2006). Singlet excited-state behavior of uracil and thymine in aqueous solution: A combined experimental and computational study of 11 uracil derivatives. J. Am. Chem. Soc..

[25] Mercier Y., Santoro F., Reguero M., Improta R. (2008). The decay from the dark np* excited state in uracil: An integrated CASPT2/CASSCF and PCM/TD-DFT study in the gas phase and in water. J. Phys. Chem. B.

[26] Zechmann G., Barbatti M. (2008). Photophysics and deactivation pathways of thymine. J. Phys. Chem. A.

[27] Blancafort L., Robb M.A. (2004). Key role of a threefold state crossing in the ultrafast decay of electronically excited cytosine. J. Phys. Chem. A.

[28] Ismail N., Blancafort L., Olivucci M., Kohler B., Robb M.A. (2002). Ultrafast decay of electronically excited singlet cytosine via pp* to n_o_p* state switch. J. Am. Chem. Soc..

[29] Blancafort L. (2007). Energetics of cytosine singlet excited-state decay paths—A difficult case for CASSCF and CASPT2. Photochem. Photobiol..

[30] Merchán M., Serrano-Andrés L., Robb M.A., Blancafort L. (2005). Triplet-state formation along the ultrafast decay of excited singlet cytosine. J. Am. Chem. Soc..

[31] Nakayama A., Yamazaki S., Taketsugu T. (2014). Quantum chemical investigations on the nonradiative deactivation pathways of cytosine derivatives. J. Phys. Chem. A.

[32] Nakayama A., Arai G., Yamazaki S., Taketsugu T. (2013). Solvent effects on the ultrafast nonradiative deactivation mechanisms of thymine in aqueous solution: Excited-state qm/mm molecular dynamics simulations. J. Chem. Phys..

[33] Nakayama A., Harabuchi Y., Yamazaki S., Taketsugu T. (2013). Photophysics of cytosine tautomers: New insights into the nonradiative decay mechanisms from MS-CASPT2 potential energy calculations and excited-state molecular dynamics simulations. Phys. Chem. Chem. Phys..

[34] Yamazaki S., Taketsugu T. (2012). Nonradiative deactivation mechanisms of uracil, thymine, and 5-fluorouracil: A comparative ab initio study. J. Phys. Chem. A.

[35] Szymczak J.J., Barbatti M., Hoo J.T.S., Adkins J.A., Windus T.L., Nachtigallova D., Lischka H. (2009). Photodynamics simulations of thymine: Relaxation into the first excited singlet state. J. Phys. Chem. A.

[36] Asturiol D., Lasorne B., Worth G.A., Robb M.A., Blancafort L. (2010). Exploring the sloped-to-peaked S_2_/S_1_ seam of intersection of thymine with electronic structure and direct quantum dynamics calculations. Phys. Chem. Chem. Phys..

[37] Asturiol D., Lasorne B., Robb M.A., Blancafort L. (2009). Photophysics of the pp* and np* states of thymine: MS-CASPT2 minimum-energy paths and casscf on-the-fly dynamics. J. Phys. Chem. A.

[38] Picconi D., Barone V., Lami A., Santoro F., Improta R. (2011). The interplay between ππ*/nπ* excited states in gas-phase thymine: A quantum dynamical study. ChemPhysChem.

[39] Mai S., Marquetand P., González L. (2015). A general method to describe intersystem crossing dynamics in trajectory surface hopping. Int. J. Quantum Chem..

[40] Mai S., Richter M., Marquetand P., González L. (2015). Excitation of nucleobases from a computational perspective II: Dynamics. Top. Curr. Chem..

[41] Buchner F., Nakayama A., Yamazaki S., Ritze H.-H., Lübcke A. (2015). Excited-state relaxation of hydrated thymine and thymidine measured by liquid-jet photoelectron spectroscopy: Experiment and simulation. J. Am. Chem. Soc..

[42] Roos B.O. (1987). The complete active space self-consistent field method and its applications in electronic structure calculations. Adv. Chem. Phys..

[43] Andersson K., Malmqvist P.A., Roos B.O. (1992). 2nd-order perturbation-theory with a complete active space self-consistent field reference function. J. Chem. Phys..

[44] Blancafort L. (2014). Photochemistry and photophysics at extended seams of conical intersection. ChemPhysChem.

[45] Roos B.O., Lindh R., Malmqvist P.A., Veryazov V., Widmark P.O. (2016). Multiconfigurational Quantum Chemistry.

[46] Serrano-Andrés L., Merchán M. (2005). Quantum chemistry of the excited state: 2005 overview. Theochem-J. Mol. Struct..

[47] Merchán M., Serrano-Andrés L., Olivucci M. (2005). Ab initio methods for excited states. Computational Photochemistry.

[48] Roca-Sanjuán D., Aquilante F., Lindh R. (2012). Multiconfiguration second-order perturbation theory approach to strong electron correlation in chemistry and photochemistry. Wiley Interdiscip. Rev. Comput. Mol. Sci..

[49] Giussani A., Segarra-Martí J., Nenov A., Rivalta I., Tolomelli A., Mukamel S., Garavelli M. (2016). Spectroscopic fingerprints of DNA/RNA pyrimidine nucleobases in third-order nonlinear electronic spectra. Theor. Chem. Acc..

[50] Olaso-González G., Merchán M., Serrano-Andrés L. (2009). The role of adenine excimers in the photophysics of oligonucleotides. J. Am. Chem. Soc..

[51] González-Ramírez I., Segarra-Martí J., Serrano-Andrés L., Merchán M., Rubio M., Roca-Sanjuán D. (2012). On the N1-H and N3-H bond dissociation in uracil by low energy electrons: A CASSCF/CASPT2 study. J. Chem. Theor. Comput..

[52] Francés-Monerris A., Segarra-Martí J., Merchán M., Roca-Sanjuán D. (2015). Complete-active-space second-order perturbation theory (CASPT2//CASSCF) study of the dissociative electron attachment in canonical DNA nucleobases caused by low-energy electrons (0–3 eV). J. Chem. Phys..

[53] Malone R.J., Miller A.M., Kohler B. (2003). Singlet excited-state lifetimes of cytosine derivatives measured by femtosecond transient absorption. Photochem. Photobiol..

[54] Pecourt J.M.L., Peon J., Kohler B. (2001). DNA excited-state dynamics: Ultrafast internal conversion and vibrational cooling in a series of nucleosides. J. Am. Chem. Soc..

[55] Hare P.M., Crespo-Hernández C.E., Kohler B. (2007). Internal conversion to the electronic ground state occurs via two distinct pathways for pyrimidine bases in aqueous solution. Proc. Natl. Acad. Sci. USA.

[56] Hare P.M., Middleton C.T., Mertel K.I., Herbert J.M., Kohler B. (2008). Time-resolved infrared spectroscopy of the lowest triplet state of thymine and thymidine. Chem. Phys..

[57] Chen J., Zhang Y., Kohler B. (2015). Excited states in DNA strands investigated by ultrafast laser spectroscopy. Top. Curr. Chem..

[58] Doorley G.W., Wojdyla M., Watson G.W., Towrie M., Parker A.W., Kelly J.M., Quinn S.J. (2013). Tracking DNA excited states by picosecond-time-resolved infrared spectroscopy: Signature band for a charge-transfer excited state in stacked adenine—Thymine systems. J. Phys. Chem. Lett..

[59] Quinn S., Doorley G.W., Watson G.W., Cowan A.J., George M.W., Parker A.W., Ronayne K.L., Towrie M., Kelly J.M. (2007). Ultrafast IR spectroscopy of the short-lived transients formed by UV excitation of cytosine derivatives. Chem. Commun..

[60] Chatterley A.S., West C.W., Stavros V.G., Verlet J.R.R. (2014). Time-resolved photoelectron imaging of the isolated deprotonated nucleotides. Chem. Sci..

[61] Chatterley A.S., West C.W., Roberts G.M., Stavros V.G., Verlet J.R.R. (2014). Mapping the ultrafast dynamics of adenine onto its nucleotide and oligonucleotides by time-resolved photoelectron imaging. J. Phys. Chem. Lett..

[62] Onidas D., Markovitsi D., Marguet S., Sharonov A., Gustavsson T. (2002). Fluorescence properties of DNA nucleosides and nucleotides: A refined steady-state and femtosecond investigation. J. Phys. Chem. B.

[63] Segarra-Martí J., Garavelli M., Aquilante F. (2015). Multiconfigurational second-order perturbation theory with frozen natural orbitals extended to the treatment of photochemical problems. J. Chem. Theor. Comput..

[64] Li Q., Giussani A., Segarra-Martí J., Nenov A., Rivalta I., Voityuk A.A., Mukamel S., Roca-Sanjuán D., Garavelli M., Blancafort L. (2016). Multiple decay mechanisms and 2D-UV spectroscopic fingerprints of singlet excited solvated adenine-uracil monophosphate. Chem. Eur. J..

[65] Ben-Nun M., Martínez T.J. (2000). Photodynamics of ethylene: Ab initio studies of conical intersections. Chem. Phys..

[66] Molina V., Merchán M., Roos B.O., Malmqvist P.-A. (2000). On the low-lying singlet excited states of styrene: A theoretical contribution. Phys. Chem. Chem. Phys..

[67] Richter M., Mai S., Marquetand P., Gonzalez L. (2014). Ultrafast intersystem crossing dynamics in uracil unravelled by ab initio molecular dynamics. Phys. Chem. Chem. Phys..

[68] González-Luque R., Climent T., González-Ramírez I., Merchán M., Serrano-Andrés L. (2010). Singlet-triplet states interaction regions in DNA/RNA nucleobase hypersurfaces. J. Chem. Theor. Comput..

[69] Serrano-Pérez J.J., González-Luque R., Merchán M., Serrano-Andrés L. (2007). On the intrinsic population of the lowest triplet state of thymine. J. Phys. Chem. B.

[70] El-Sayed M.A. (1963). Spin—Orbit coupling and the radiationless processes in nitrogen heterocyclics. J. Chem. Phys..

[71] Szymczak J.J., Barbatti M., Lischka H. (2011). Influence of the active space on CASSCF nonadiabatic dynamics simulations. Int. J. Quantum Chem..

[72] Plasser F., Crespo-Otero R., Pederzoli M., Pittner J., Lischka H., Barbatti M. (2014). Surface hopping dynamics with correlated single-reference methods: 9H-adenine as a case study. J. Chem. Theor. Comput..

[73] Martínez-Fernández L., Pepino A.J., Segarra-Martí J., Banyasz A., Garavelli M., Improta R. (2016). Computing the absorption and emission spectra of 5-methylcytidine in different solvents: A test-case for different solvation models. J. Chem. Theor. Comput..

[74] Szalay P.G., Watson T., Perera A., Lotrich V.F., Bartlett R.J. (2012). Benchmark studies on the building blocks of DNA. 1. Superiority of coupled cluster methods in describing the excited states of nucleobases in the Franck-Condon region. J. Phys. Chem. A.

[75] Altavilla S.F., Segarra-Martí J., Nenov A., Conti I., Rivalta I., Garavelli M. (2015). Deciphering the photochemical mechanisms describing the UV-induced processes occurring in solvated guanine monophosphate. Front. Chem..

[76] Conti I., Altoe P., Stenta M., Garavelli M., Orlandi G. (2010). Adenine deactivation in DNA resolved at the CASPT2//CASSCF/AMBER level. Phys. Chem. Chem. Phys..

[77] Altoé P., Stenta M., Bottoni A., Garavelli M. (2007). A tunable QM/MM approach to chemical reactivity, structure and physico-chemical properties prediction. Theor. Chem. Acc..

[78] De Vico L., Olivucci M., Lindh R. (2005). New general tools for constrained geometry optimizations. J. Chem. Theor. Comput..

[79] Canuel C., Mons M., Piuzzi F., Tardivel B., Dimicoli I., Elhanine M. (2005). Excited states dynamics of DNA and RNA bases: Characterization of a stepwise deactivation pathway in the gas phase. J. Chem. Phys..

[80] Levine B.G., Ko C., Quenneville J., MartÍnez T.J. (2006). Conical intersections and double excitations in time-dependent density functional theory. Mol. Phys..

[81] Serrano-Andrés L., Merchán M., Lindh R. (2005). Computation of conical intersections by using perturbation techniques. J. Chem. Phys..

[82] Granovsky A.A. (2011). Extended multi-configuration quasi-degenerate perturbation theory: The new approach to multi-state multi-reference perturbation theory. J. Chem. Phys..

[83] Gozem S., Melaccio F., Lindh R., Krylov A.I., Granovsky A.A., Angeli C., Olivucci M. (2013). Mapping the excited state potential energy surface of a retinal chromophore model with multireference and equation-of-motion coupled-cluster methods. J. Chem. Theor. Comput..

[84] Gozem S., Huntress M., Schapiro I., Lindh R., Granovsky A.A., Angeli C., Olivucci M. (2012). Dynamic electron correlation effects on the ground state potential energy surface of a retinal chromophore model. J. Chem. Theor. Comput..

[85] Shiozaki T., Woywod C., Werner H.-J. (2013). Pyrazine excited states revisited using the extended multi-state complete active space second-order perturbation method. Phys. Chem. Chem. Phys..

[86] Shiozaki T., Werner H.-J. (2013). Multireference explicitly correlated F12 theories. Mol. Phys..

[87] Shiozaki T., Gyrffy W., Celani P., Werner H.-J. (2011). Communication: Extended multi-state complete active space second-order perturbation theory: Energy and nuclear gradients. J. Chem. Phys..

[88] Vlaisavljevich B., Shiozaki T. (2016). Nuclear energy gradients for internally contracted complete active space second-order perturbation theory: Multistate extensions. J. Chem. Theor. Comput..

[89] Liu L., Liu J., Martinez T.J. (2016). Dynamical correlation effects on photoisomerization: Ab initio multiple spawning dynamics with MS-CASPT2 for a model trans-protonated schiff base. J. Phys. Chem. B.

[90] Mai S., Marquetand P., González L. (2016). Intersystem crossing pathways in the noncanonical nucleobase 2-thiouracil: A time-dependent picture. J. Phys. Chem. Lett..

[91] Tao H., Levine B.G., Martínez T.J. (2009). Ab initio multiple spawning dynamics using multi-state second-order perturbation theory. J. Phys. Chem. A.

[92] Frutos L.M., Andruniów T., Santoro F., Ferré N., Olivucci M. (2007). Tracking the excited-state time evolution of the visual pigment with multiconfigurational quantum chemistry. Proc. Natl. Acad. Sci. USA.

[93] Mai S., Richter M., Marquetand P., González L. (2016). The DNA nucleobase thymine in motion—Intersystem crossing simulated with surface hopping. Chem. Phys..

[94] Nenov A., Giussani A., Segarra-martí J., Vishal K., Rivalta I., Cerullo G., Mukamel S. (2015). Modeling the high-energy electronic state manifold of adenine: Calibration for nonlinear electronic spectroscopy. J. Chem. Phys..

[95] Barbatti M., Aquino A.J.A., Szymczak J.J., Nachtigallova D., Hobza P., Lischka H. (2010). Relaxation mechanisms of UV-photoexcited DNA and RNA nucleobases. Proc. Natl. Acad. Sci. USA.

[96] Aquilante F., de Vico L., Ferré N., Ghigo G., Malmqvist P.A., Neogrady P., Pedersen T.B., Pitonak M., Reiher M., Roos B.O. (2010). Software news and update MOLCAS 7: The next generation. J. Computat. Chem..

[97] Aquilante F., Autschbach J., Carlson R., Chibotaru L., Delcey M.G., De Vico L., Fernández Galvan I., Ferré N., Frutos L.M., Gagliardi L. (2016). MOLCAS 8: New capabilities for multiconfigurational quantum chemical calculations across the periodic table. J. Comput. Chem..

[98] Pierloot K., Dumez B., Widmark P.O., Roos B.O. (1995). Density matrix averaged atomic natural orbital (ANO) basis sets for correlated molecular wave functions. IV. Medium size basis sets for the atoms h-kr. Theor. Chim. Acta.

[99] Widmark P.O., Persson B.J., Roos B.O. (1991). Density matrix averaged atomic natural orbital (ANO) basis sets for correlated molecular wave functions. II. Second row atoms. Theor. Chim. Acta.

[100] Forsberg N., Malmqvist P.A. (1997). Multiconfiguration perturbation theory with imaginary level shift. Chem. Phys. Lett..

[101] Finley J., Malmqvist P.A., Roos B.O., Serrano-Andrés L. (1998). The multi-state CASPT2 method. Chem. Phys. Lett..

[102] Delcey M.G., Pedersen T.B., Aquilante F., Lindh R. (2015). Analytical gradients of the state-average complete active space self-consistent field method with density fitting. J. Chem. Phys..

[103] Frisch M.J., Trucks G.W., Schlegel H.B., Scuseria G.E., Robb M.A., Cheeseman J.R., Scalmani G., Barone V., Mennucci B., Petersson G.A. (2009). Gaussian 09, revision b.01.

[104] Bearpark M.J., Robb M.A., Bernhard Schlegel H. (1994). A direct method for the location of the lowest energy point on a potential surface crossing. Chem. Phys. Lett..

[105] Nenov A., Segarra-Martí J., Giussani A., Conti I., Rivalta I., Dumont E., Jaiswal V.K., Altavilla S.F., Mukamel S., Garavelli M. (2015). Fd 177: Probing deactivation pathways of DNA nucleobases by two-dimensional electronic spectroscopy: First principles simulations. Faraday Discuss..

[106] West B.A., Moran A.M. (2012). Two-dimensional electronic spectroscopy in the ultraviolet wavelength range. J. Phys. Chem. Lett..

[107] West B.A., Womick J.M., Moran A.M. (2011). Probing ultrafast dynamics in adenine with mid-UV four-wave mixing spectroscopies. J. Phys. Chem. A.

