# Supplementary material for: Assessment of the Potential Energy Hypersurfaces in Thymine within Multiconfigurational Theory: CASSCF vs. CASPT2
Source: Molecules. 2016 Dec 3;21(12):1666. doi: 10.3390/molecules21121666 (PMC6274573; doi:10.3390/molecules21121666)
Supplement: Supplementary file 1 [file molecules-21-01666-s001.pdf]

Supplementary Materials: Assessment of the Potential Energy Hypersurfaces in Thymine within Multiconfigurational Theory: CASSCF vs. CASPT2

Javier Segarra-Martí, Antonio Francés-Monerris, Daniel Roca-Sanjuán and Manuela Merchán

Table S1. Cartesian coordinates (in Å) for the S_0 equilibrium geometry of thymine obtained at the CASSCF(10,8)/ANO-L O [4s,3p,2d]/H [2s,1p] level of theory.

N1	−0.73236	1.03148	1.49914
N2	−1.11813	−1.24096	1.49935
C1	−1.66309	0.01578	1.49991
C2	0.24745	−1.47775	1.50039
C3	1.15475	−0.48408	1.49951
C4	2.64189	−0.70221	1.50020
C5	0.64871	0.90254	1.49944
O1	1.35213	1.87615	1.50061
O2	−2.84376	0.21964	1.50080
H1	0.52915	−2.51706	1.50181
H2	−1.10159	1.96179	1.49972
H3	−1.76809	−1.99718	1.49847
H4	2.87135	−1.76516	1.49406
H5	3.09504	−0.25093	2.38065
H6	3.09677	−0.24069	0.62605

Table S2. Cartesian coordinates (in Å) for the optimized (loose threshold, see Table 1 in the paper) lowest-lying $\pi\pi^*$ state of thymine obtained at the CASSCF(10,8)/6-31G level of theory.

N1	−0.748161	1.016722	1.498784
N2	−1.139687	−1.244840	1.500266
C1	−1.582047	0.006108	1.500451
C2	0.256435	−1.534070	1.498306
C3	1.153668	−0.444665	1.498984
C4	2.635614	−0.694622	1.501156
C5	0.717380	0.880800	1.498672
O1	1.346630	1.975417	1.498663
O2	−2.908101	0.214432	1.502728
H1	0.523367	−2.564640	1.495879
H2	−1.094021	1.952964	1.498425
H3	−1.810653	−1.978991	1.502090
H4	2.857111	−1.755335	1.495437
H5	3.099136	−0.254279	2.376348
H6	3.103563	−0.243614	0.633922

Table S3. Cartesian coordinates (in Å) for the optimized (loose threshold, see Table 1 in the paper) lowest-lying $\pi\pi^*$ state of thymine obtained at the CASSCF(10,8)/6-31G* level of theory.

C1	−1.640670	0.052223	1.500509
C2	0.205081	−1.574175	1.500060
C3	1.172520	−0.417966	1.498662
C4	2.648365	−0.680588	1.501179
C5	0.677706	0.881297	1.497456
N1	−0.743646	1.045139	1.497608
N2	−1.088550	−1.241826	1.501453
O1	1.320147	1.945944	1.496614
O2	−2.847164	0.145958	1.502249
H1	0.468251	−2.608750	1.499179
H2	−1.084961	1.981164	1.496764
H3	−1.794899	−1.952369	1.503335
H4	2.859155	−1.746186	1.487247
H5	3.126706	−0.261449	2.381829
H6	3.132191	−0.237027	0.635967

Table S4. Cartesian coordinates (in Å) for the optimized (tight threshold, see Table 1 in the paper) lowest-lying $\pi\pi^*$ state of thymine obtained at the CASSCF(10,8)/6-31G level of theory.

N1	−0.748162	1.016732	1.499197
N2	−1.139706	−1.244856	1.501577
C1	−1.582054	0.006102	1.499714
C2	0.256420	−1.534110	1.505358
C3	1.153644	−0.444712	1.500939
C4	2.635602	−0.694612	1.500646
C5	0.717368	0.880775	1.499641
O1	1.346749	1.975321	1.498688
O2	−2.908122	0.214408	1.498516
H1	0.523346	−2.564623	1.493351
H2	−1.093967	1.952998	1.497798
H3	−1.810692	−1.978988	1.502688
H4	2.857168	−1.755282	1.492503
H5	3.100573	−0.256016	2.375936
H6	3.102065	−0.241750	0.633560

Table S5. Cartesian coordinates (in Å) for the optimized (tight threshold, see Table 1 in the paper) lowest-lying $\pi\pi^*$ state of thymine obtained at the CASSCF(10,8)/6-31G* level of theory.

C1	−1.049932	−0.073327	2.098389
C2	0.245551	−1.511451	0.736585
C3	1.303656	−0.521388	0.648616
C4	2.115322	−0.533174	1.972184
C5	0.698482	0.825622	0.562333
N1	−0.495812	0.928701	1.372183
N2	−0.665527	−1.392503	1.666413
O1	1.103502	1.839485	0.074738
O2	−1.894349	0.027719	2.937377
H1	0.153022	−2.382155	0.101872
H2	−0.862774	1.840325	1.551284
H3	−1.327552	−2.108862	1.901211
H4	2.501288	−1.527564	2.173662
H5	1.608494	−0.188362	2.880323
H6	2.976861	0.108321	1.822941

Table S6. Cartesian coordinates (in Å) for the final structure in the minimum energy path along lowest-lying $\pi\pi^*$ state of thymine obtained at the CASSCF(10,8)/6-31G level of theory.

N1	−0.747356	1.017398	1.496451
N2	−1.140231	−1.243928	1.500213
C1	−1.581854	0.007309	1.500588
C2	0.255695	−1.534106	1.487781
C3	1.153610	−0.445181	1.494819
C4	2.635350	−0.695942	1.500619
C5	0.718054	0.880495	1.495913
O1	1.347974	1.974654	1.495449
O2	−2.907758	0.216532	1.505853
H1	0.522104	−2.564439	1.517722
H2	−1.092489	1.953905	1.500558
H3	−1.811648	−1.977699	1.497786
H4	2.856457	−1.756572	1.481047
H5	3.095393	−0.267742	2.383733
H6	3.106932	−0.233297	0.641579

Table S7. Cartesian coordinates (in Å) for the final structure in the minimum energy path along lowest-lying $\pi\pi^*$ state of thymine obtained at the CASSCF(10,8)/6-31G* level of theory.

C1	−1.075576	−0.068772	2.076168
C2	0.230907	−1.485493	0.708127
C3	1.298055	−0.502678	0.654646
C4	2.123420	−0.576837	1.967351
C5	0.725304	0.857016	0.619493
N1	−0.504395	0.942587	1.373626
N2	−0.682882	−1.382108	1.638191
O1	1.182164	1.888455	0.221719
O2	−1.944633	0.023930	2.890559
H1	0.134961	−2.337445	0.049267
H2	−0.851038	1.854914	1.586222
H3	−1.345545	−2.101660	1.861371
H4	2.489455	−1.584737	2.136305
H5	1.633322	−0.246264	2.889605
H6	2.996712	0.050480	1.827464

Table S8. Cartesian coordinates (in Å) for the optimized (loose threshold, see Table 1 in the paper) lowest-lying $\pi\pi^*$ state of thymine obtained at the CASSCF(10,8)/ANO-S O [3s,2p,1d]/H [2s,1p] level of theory.

N1	−0.747174	1.054864	1.499875
N2	−1.083338	−1.243706	1.499437
C1	−1.636917	0.054815	1.500039
C2	0.208741	−1.572229	1.498482
C3	1.172133	−0.418599	1.499223
C4	2.648464	−0.685109	1.500652
C5	0.673496	0.887792	1.500022
O1	1.322075	1.941759	1.500912
O2	−2.840063	0.144076	1.500561
H1	0.474030	−2.612076	1.497172
H2	−1.095775	1.993391	1.500988
H3	−1.791165	−1.960518	1.499790
H4	2.851316	−1.756118	1.499836
H5	3.126291	−0.249288	2.379653
H6	3.128119	−0.247667	0.623471

Table S9. Cartesian coordinates (in Å) for the optimized (loose threshold, see Table 1 in the paper) lowest-lying $\pi\pi^*$ state of thymine obtained at the CASSCF(10,8)/ANO-L O [3s,2p,1d]/H [2s,1p] level of theory.

N1	−0.746602	1.053513	1.499966
N2	−1.082995	−1.243145	1.499367
C1	−1.635769	0.053932	1.499893
C2	0.208559	−1.571758	1.498985
C3	1.171572	−0.418109	1.499314
C4	2.646579	−0.684489	1.500477
C5	0.673177	0.885652	1.499968
O1	1.323232	1.940481	1.500697
O2	−2.839563	0.144526	1.500147
H1	0.474310	−2.611708	1.498247
H2	−1.094632	1.992090	1.501348
H3	−1.791130	−1.959385	1.499352
H4	2.850165	−1.755112	1.499052
H5	3.125791	−0.248909	2.379204
H6	3.127538	−0.246192	0.624094

Table S10. Cartesian coordinates (in Å) for the optimized (loose threshold, see Table 1 in the paper) lowest-lying $\pi\pi^*$ state of thymine obtained at the CASSCF(10,8)/ANO-L O [4s,3p,2d]/H [2s,1p] level of theory.

N1	−0.745315	1.051151	1.498593
N2	−1.079361	−1.240166	1.500562
C1	−1.631520	0.053173	1.499967
C2	0.208533	−1.569431	1.500294
C3	1.168209	−0.419738	1.499261
C4	2.640417	−0.683708	1.500555
C5	0.670355	0.883044	1.498630
O1	1.321554	1.932252	1.498533
O2	−2.832550	0.142505	1.500664
H1	0.472829	−2.602972	1.500228
H2	−1.089351	1.982996	1.498676
H3	−1.782154	−1.950644	1.501444
H4	2.848299	−1.749542	1.490847
H5	3.118335	−0.257286	2.378740
H6	3.121952	−0.240249	0.633117

Table S11. Cartesian coordinates (in Å) for the optimized (tight threshold, see Table 1 in the paper) lowest-lying $\pi\pi^*$ state of thymine obtained at the CASSCF(10,8)/ANO-S O [3s,2p,1d]/H [2s,1p] level of theory.

N1	−0.467661	0.966795	1.407201
N2	−0.701627	−1.364907	1.595269
C1	−1.044177	−0.056695	2.091702
C2	0.208809	−1.457759	0.660433
C3	1.307190	−0.505691	0.640333
C4	2.094970	−0.619953	1.977429
C5	0.752749	0.868943	0.640006
O1	1.224981	1.893353	0.250418
O2	−1.874621	0.032424	2.941641
H1	0.088339	−2.287169	−0.035501
H2	−0.780894	1.883595	1.668297
H3	−1.399552	−2.070701	1.770457
H4	2.411216	−1.650492	2.139025
H5	1.591664	−0.276438	2.892185
H6	2.998845	−0.023917	1.861217

Table S12. Cartesian coordinates (in Å) for the optimized (tight threshold, see Table 1 in the paper) lowest-lying $\pi\pi^*$ state of thymine obtained at the CASSCF(10,8)/ANO-L O [3s,2p,1d]/H [2s,1p] level of theory.

N1	−0.530895	0.918350	1.395213
N2	−0.645763	−1.416029	1.641044
C1	−1.078810	−0.237388	0.937805
C2	0.299313	−1.296530	2.536029
C3	1.339463	−0.302363	2.338033
C4	2.098777	−0.624293	1.019080
C5	0.715164	1.021661	2.116595
O1	1.141946	2.121503	2.299782
O2	−1.948784	−0.342154	0.129640
H1	0.248690	−1.991622	3.372603
H2	−0.908220	1.757491	0.994000
H3	−1.305762	−2.177689	1.618688
H4	1.549124	−0.467058	0.080052
H5	2.459491	−1.652515	1.026343
H6	2.976499	0.020021	0.995203

Table S13. Cartesian coordinates (in Å) for the optimized (tight threshold, see Table 1 in the paper) lowest-lying *ππ** state of thymine obtained at the CASSCF(10,8)/ANO-L O [4s,3p,2d]/H [2s,1p] level of theory.

N1	−0.520728	0.916219	1.351899
N2	−0.648666	−1.403922	1.675721
C1	−1.040470	−0.092862	2.097875
C2	0.247667	−1.508800	0.730373
C3	1.291321	−0.510495	0.628190
C4	2.101809	−0.518073	1.960341
C5	0.688269	0.840342	0.574778
O1	1.107694	1.861430	0.124398
O2	−1.854069	0.011091	2.955367
H1	0.170891	−2.388351	0.107193
H2	−0.889543	1.821251	1.543279
H3	−1.290587	−2.129628	1.923475
H4	2.457935	−1.521596	2.168489
H5	1.615820	−0.149487	2.868926
H6	2.972889	0.104269	1.789807

Table S14. Cartesian coordinates (in Å) for the final structure in the minimum energy path along lowest-lying *ππ** state of thymine obtained at the CASSCF(10,8)/ANO-S O [3s,2p,1d]/H [2s,1p] level of theory.

N1	−0.536293	0.925242	1.401624
N2	−0.648529	−1.413946	1.629296
C1	−1.097095	−0.226774	0.948352
C2	0.293096	−1.295157	2.529889
C3	1.338150	−0.302666	2.336195
C4	2.109933	−0.635570	1.026831
C5	0.723913	1.023088	2.100343
O1	1.167477	2.120214	2.253888
O2	−1.988344	−0.323194	0.163254
H1	0.237337	−1.990301	3.366918
H2	−0.908606	1.766013	0.999937
H3	−1.308607	−2.175550	1.605431
H4	1.573476	−0.474601	0.081004
H5	2.461629	−1.667186	1.041818
H6	2.992695	0.001774	1.015332

Table S15. Cartesian coordinates (in Å) for the final structure in the minimum energy path along lowest-lying $\pi\pi^*$ state of thymine obtained at the CASSCF(10,8)/ANO-L O [3s,2p,1d]/H [2s,1p] level of theory.

N1	−0.530889	0.925554	1.604495
N2	−0.652204	−1.410464	1.364067
C1	−1.093139	−0.226434	2.053560
C2	0.289860	−1.291545	0.465271
C3	1.334780	−0.302246	0.664186
C4	2.107068	−0.639626	1.971649
C5	0.726172	1.023663	0.902679
O1	1.175651	2.119989	0.754930
O2	−1.982759	−0.324036	2.841053
H1	0.233834	−1.982544	−0.374175
H2	−0.906747	1.766358	2.003420
H3	−1.311905	−2.172237	1.390777
H4	1.569507	−0.487244	2.918029
H5	2.987454	0.001294	1.989042
H6	2.463553	−1.669096	1.951128

Table S16. Cartesian coordinates (in Å) for the final structure in the minimum energy path along lowest-lying $\pi\pi^*$ state of thymine obtained at the CASSCF(10,8)/ANO-L O [4s,3p,2d]/H [2s,1p] level of theory.

N1	−0.516959	0.933038	1.381137
N2	−0.635189	−1.395714	1.643955
C1	−1.066553	−0.216148	0.938195
C2	0.312914	−1.278226	2.524026
C3	1.349086	−0.295222	2.334024
C4	2.093371	−0.657275	1.021637
C5	0.709240	1.032299	2.122623
O1	1.082919	2.143642	2.330252
O2	−1.935594	−0.314314	0.135782
H1	0.257707	−1.969603	3.355367
H2	−0.899017	1.766791	0.991321
H3	−1.290654	−2.154732	1.625782
H4	1.554029	−0.529798	0.071805
H5	2.417071	−1.693334	1.056929
H6	2.977860	−0.040014	0.967279

Table S17. Cartesian coordinates (in Å) for the optimized (loose threshold, see Table 1 in the paper) lowest-lying $\pi\pi^*$ state of thymine obtained at the CASSCF(10,11)/6-31G level of theory.

N1	−0.749638	1.019130	1.498375
N2	−1.139395	−1.246037	1.502600
C1	−1.588147	0.008692	1.499949
C2	0.255466	−1.533163	1.497814
C3	1.153640	−0.446831	1.499386
C4	2.635305	−0.696798	1.501130
C5	0.718911	0.879927	1.499158
O1	1.353473	1.980802	1.499482
O2	−2.909015	0.207969	1.498806
H1	0.523195	−2.563798	1.492639
H2	−1.090133	1.957214	1.497002
H3	−1.809234	−1.981368	1.508374
H4	2.858752	−1.757019	1.492606
H5	3.096181	−0.256354	2.377276
H6	3.100871	−0.240978	0.635514

Table S18. Cartesian coordinates (in Å) for the optimized (loose threshold, see Table 1 in the paper) lowest-lying $\pi\pi^*$ state of thymine obtained at the CASSCF(10,11)/6-31G* level of theory.

C1	−1.058352	−0.052050	2.088571
C2	0.216830	−1.466670	0.682753
C3	1.307603	−0.510248	0.650286
C4	2.109398	−0.614397	1.977992
C5	0.748957	0.859108	0.626689
N1	−0.466679	0.963382	1.407301
N2	−0.701832	−1.365772	1.608587
O1	1.207570	1.883933	0.214098
O2	−1.908805	0.038603	2.921821
H1	0.089330	−2.296291	−0.002770
H2	−0.789739	1.879006	1.644674
H3	−1.405918	−2.062219	1.779816
H4	2.434795	−1.635777	2.150859
H5	1.618651	−0.268569	2.893991
H6	3.008425	−0.020654	1.855443

Table S19. Cartesian coordinates (in Å) for the optimized (tight threshold, see Table 1 in the paper) lowest-lying $\pi\pi^*$ state of thymine obtained at the CASSCF(10,11)/6-31G level of theory.

N1	−0.749686	1.019027	1.500020
N2	−1.139358	−1.246192	1.499674
C1	−1.588088	0.008507	1.499822
C2	0.255580	−1.533300	1.499706
C3	1.153610	−0.446857	1.499932
C4	2.635322	−0.696519	1.500289
C5	0.718765	0.879852	1.500176
O1	1.353421	1.980690	1.500464
O2	−2.909037	0.207774	1.499781
H1	0.523323	−2.563945	1.499297
H2	−1.090287	1.957071	1.500124
H3	−1.809229	−1.981524	1.499572
H4	2.859020	−1.756715	1.496386
H5	3.097758	−0.251649	2.373340
H6	3.099119	−0.244833	0.631527

Table S20. Cartesian coordinates (in Å) for the optimized (tight threshold, see Table 1 in the paper) lowest-lying $\pi\pi^*$ state of thymine obtained at the CASSCF(10,11)/6-31G* level of theory.

C1	−1.065051	−0.048263	2.087945
C2	0.219939	−1.475744	0.680749
C3	1.302428	−0.509099	0.650388
C4	2.114221	−0.618443	1.971492
C5	0.748001	0.860728	0.632070
N1	−0.467845	0.966016	1.412903
N2	−0.702254	−1.362358	1.606920
O1	1.210897	1.885427	0.222330
O2	−1.920773	0.047010	2.914727
H1	0.097971	−2.313955	0.005579
H2	−0.789517	1.881516	1.651286
H3	−1.409217	−2.055388	1.774686
H4	2.440421	−1.637439	2.149120
H5	1.628546	−0.278364	2.891333
H6	3.002465	−0.010256	1.848585

Table S21. Cartesian coordinates (in Å) for the final structure in the minimum energy path along lowest-lying $\pi\pi^*$ state of thymine obtained at the CASSCF(10,11)/6-31G level of theory.

N1	−0.749317	1.018901	1.535188
N2	−1.138809	−1.245412	1.470609
C1	−1.587739	0.008916	1.496418
C2	0.255882	−1.532487	1.485489
C3	1.153846	−0.445756	1.513164
C4	2.635107	−0.697664	1.496020
C5	0.718348	0.880597	1.539885
O1	1.351500	1.982317	1.558903
O2	−2.908278	0.208537	1.480599
H1	0.524454	−2.562687	1.461596
H2	−1.090334	1.956539	1.552997
H3	−1.808541	−1.980295	1.440292
H4	2.861961	−1.741996	1.675898
H5	3.127917	−0.101367	2.252809
H6	3.064235	−0.416756	0.540244

Table S22. Cartesian coordinates (in Å) for the final structure in the minimum energy path along lowest-lying $\pi\pi^*$ state of thymine obtained at the CASSCF(10,11)/6-31G* level of theory.

C1	−1.099774	−0.220285	2.059907
C2	0.289515	−1.298010	0.477275
C3	1.336469	−0.310616	0.667427
C4	2.115792	−0.639706	1.970893
C5	0.726966	1.016773	0.889906
N1	−0.526265	0.925325	1.607539
N2	−0.658549	−1.409673	1.372895
O1	1.169655	2.116256	0.722088
O2	−1.993878	−0.315104	2.846184
H1	0.220340	−1.987022	−0.355998
H2	−0.905616	1.768324	1.987261
H3	−1.329660	−2.157405	1.392546
H4	1.590524	−0.503509	2.921973
H5	2.986832	0.005648	1.992160
H6	2.487882	−1.659608	1.948056

Table S23. Cartesian coordinates (in Å) for the optimized (loose threshold, see Table 1 in the paper) lowest-lying $\pi\pi^*$ state of thymine obtained at the CASSCF(10,11)/ANO-S O [3s,2p,1d]/H [2s,1p] level of theory.

N1	−0.749026	1.058621	1.472823
N2	−1.082013	−1.244629	1.492005
C1	−1.639307	0.060020	1.484977
C2	0.216791	−1.572427	1.511089
C3	1.167382	−0.430436	1.507364
C4	2.645724	−0.687476	1.498256
C5	0.674781	0.883994	1.497443
O1	1.333148	1.938638	1.499454
O2	−2.843165	0.144186	1.495232
H1	0.481950	−2.612384	1.520962
H2	−1.093088	1.997475	1.541956
H3	−1.791186	−1.961064	1.490147
H4	2.856918	−1.756620	1.496130
H5	3.120726	−0.244299	2.374126
H6	3.110599	−0.242211	0.618147

Table S24. Cartesian coordinates (in Å) for the optimized (loose threshold, see Table 1 in the paper) lowest-lying $\pi\pi^*$ state of thymine obtained at the CASSCF(10,11)/ANO-L O [3s,2p,1d]/H [2s,1p] level of theory.

N1	−0.515146	0.941762	1.355899
N2	−0.674434	−1.384859	1.662250
C1	−1.081405	−0.065275	2.073736
C2	0.234805	−1.498025	0.723645
C3	1.301224	−0.514502	0.653665
C4	2.133716	−0.565160	1.965754
C5	0.714375	0.843525	0.604155
O1	1.172440	1.863876	0.183259
O2	−1.940171	0.040065	2.894138
H1	0.117252	−2.351626	0.055670
H2	−0.830098	1.862943	1.605064
H3	−1.347737	−2.105174	1.881568
H4	2.445062	−1.586684	2.183288
H5	1.640914	−0.164074	2.862025
H6	3.039434	0.014595	1.795992

Table S25. Cartesian coordinates (in Å) for the optimized (loose threshold, see Table 1 in the paper) lowest-lying $\pi\pi^*$ state of thymine obtained at the CASSCF(10,11)/ANO-L O [4s,3p,2d]/H [2s,1p] level of theory.

N1	−0.499050	0.949016	1.373231
N2	−0.682703	−1.378475	1.634304
C1	−1.055727	−0.065568	2.085781
C2	0.222294	−1.478136	0.697112
C3	1.297956	−0.505697	0.657810
C4	2.120689	−0.593285	1.973661
C5	0.725084	0.856948	0.621123
O1	1.183563	1.875400	0.214548
O2	−1.887556	0.034062	2.930396
H1	0.095967	−2.306191	0.012261
H2	−0.822472	1.861017	1.613093
H3	−1.375889	−2.079833	1.814816
H4	2.423861	−1.614311	2.180220
H5	1.638282	−0.204569	2.877893
H6	3.025934	−0.018990	1.813861

Table S26. Cartesian coordinates (in Å) for the optimized (tight threshold, see Table 1 in the paper) lowest-lying $\pi\pi^*$ state of thymine obtained at the CASSCF(10,11)/ANO-S O [3s,2p,1d]/H [2s,1p] level of theory.

N1	−0.508659	0.960374	1.369425
N2	−0.696479	−1.376269	1.613494
C1	−1.073092	−0.062397	2.072270
C2	0.221892	−1.468853	0.679611
C3	1.307109	−0.499184	0.659237
C4	2.123575	−0.607691	1.979296
C5	0.737475	0.868685	0.645384
O1	1.222154	1.895571	0.278995
O2	−1.911341	0.033700	2.912874
H1	0.102259	−2.298536	−0.017457
H2	−0.815105	1.877622	1.640767
H3	−1.402956	−2.080751	1.768762
H4	2.396327	−1.643116	2.178350
H5	1.656954	−0.210092	2.890112
H6	3.050120	−0.057676	1.828993

Table S27. Cartesian coordinates (in Å) for the optimized (tight threshold, see Table 1 in the paper) lowest-lying $\pi\pi^*$ state of thymine obtained at the CASSCF(10,11)/ANO-L O [3s,2p,1d]/H [2s,1p] level of theory.

N1	−0.546553	0.951580	1.359214
N2	−0.676320	−1.365906	1.659349
C1	−1.107933	−0.051061	2.068936
C2	0.241708	−1.467476	0.722871
C3	1.319820	−0.518644	0.666038
C4	2.164571	−0.590382	1.966505
C5	0.678350	0.804128	0.603633
O1	1.129021	1.807895	0.141628
O2	−1.980846	0.046228	2.880227
H1	0.134253	−2.302292	0.033923
H2	−0.860804	1.873299	1.614928
H3	−1.361598	−2.081797	1.862508
H4	2.458821	−1.611012	2.210528
H5	1.775951	−0.192355	2.905109
H6	3.041789	0.029181	1.804715

Table S28. Cartesian coordinates (in Å) for the optimized (tight threshold, see Table 1 in the paper) lowest-lying $\pi\pi^*$ state of thymine obtained at the CASSCF(10,11)/ANO-L O [4s,3p,2d]/H [2s,1p] level of theory.

N1	−0.498369	0.932678	1.362839
N2	−0.677935	−1.388658	1.653035
C1	−1.045183	−0.077188	2.093442
C2	0.233862	−1.495564	0.724802
C3	1.315073	−0.544996	0.643994
C4	2.114462	−0.562416	1.979725
C5	0.704556	0.838980	0.597756
O1	1.163768	1.867275	0.152563
O2	−1.868548	0.039891	2.940797
H1	0.102604	−2.329070	0.044985
H2	−0.829696	1.844642	1.585182
H3	−1.369249	−2.089545	1.829552
H4	2.444731	−1.570683	2.206784
H5	1.614708	−0.166089	2.869600
H6	3.005445	0.032130	1.815056

Table S29. Cartesian coordinates (in Å) for the final structure in the minimum energy path along lowest-lying $\pi\pi^*$ state of thymine obtained at the CASSCF(10,11)/ANO-S O [3s,2p,1d]/H [2s,1p] level of theory.

N1	−0.537580	0.914455	1.602826
N2	−0.668169	−1.419183	1.364761
C1	−1.091694	−0.233341	2.066977
C2	0.279651	−1.312853	0.459495
C3	1.332452	−0.329557	0.661739
C4	2.115445	−0.612203	1.968451
C5	0.725759	0.994355	0.893737
O1	1.202018	2.083343	0.722450
O2	−1.949786	−0.325917	2.872970
H1	0.204937	−1.997387	−0.383023
H2	−0.865220	1.753000	2.054807
H3	−1.359403	−2.154235	1.356730
H4	1.572047	−0.398824	2.900071
H5	3.001963	0.015666	1.956566
H6	2.447812	−1.645933	2.001556

Table S30. Cartesian coordinates (in Å) for the final structure in the minimum energy path along lowest-lying $\pi\pi^*$ state of thymine obtained at the CASSCF(10,11)/ANO-L O [3s,2p,1d]/H [2s,1p] level of theory.

N1	−0.525471	0.925708	1.605588
N2	−0.659030	−1.412310	1.369581
C1	−1.079658	−0.226999	2.071278
C2	0.289562	−1.303256	0.467220
C3	1.338977	−0.314734	0.664951
C4	2.105976	−0.636451	1.979005
C5	0.717676	1.015157	0.875756
O1	1.151910	2.109521	0.674218
O2	−1.939555	−0.321731	2.891274
H1	0.216819	−1.988419	−0.377350
H2	−0.884988	1.767522	2.019131
H3	−1.350250	−2.147410	1.363183
H4	1.579779	−0.455570	2.925602
H5	2.996988	−0.009815	1.990421
H6	2.451499	−1.669824	1.980250

Table S31. Cartesian coordinates (in Å) for the final structure in the minimum energy path along lowest-lying $\pi\pi^*$ state of thymine obtained at the CASSCF(10,11)/ANO-L O [4s,3p,2d]/H [2s,1p] level of theory.

N1	−0.546011	0.922302	1.583824
N2	−0.649774	−1.417355	1.381375
C1	−1.095815	−0.227747	2.055433
C2	0.287890	−1.298417	0.477063
C3	1.330274	−0.308277	0.671846
C4	2.119042	−0.636891	1.970442
C5	0.718731	1.017587	0.899956
O1	1.172164	2.109386	0.753960
O2	−1.974017	−0.318409	2.852052
H1	0.215415	−1.970053	−0.367558
H2	−0.907660	1.758682	1.988495
H3	−1.323995	−2.158695	1.385315
H4	1.598839	−0.462869	2.918231
H5	3.003883	−0.011133	1.966922
H6	2.461271	−1.666721	1.962756

Table S32. Cartesian coordinates (in Å) for the S_0 equilibrium structure of thymine obtained at the CASPT2(10,8)/6-31G* level of theory.

N	−0.72470555	1.02175750	1.49944814
N	−1.11596200	−1.24620432	1.49951003
C	−1.67828435	0.01647030	1.49989427
C	0.24450625	−1.48556401	1.49965855
C	1.15577542	−0.48243159	1.49946995
C	2.63737045	−0.69802706	1.50037420
C	0.66909810	0.89701956	1.49972828
O	1.38681497	1.89489198	1.50040248
O	−2.88603454	0.21847745	1.50046510
H	0.52892380	−2.53279406	1.49962611
H	−1.09004557	1.97117917	1.50009958
H	−1.78090162	−2.01045844	1.49966331
H	2.87697051	−1.76455313	1.49573051
H	3.09242349	−0.23824200	2.38158700
H	3.09428364	−0.23013336	0.62445649

Table S33. Cartesian coordinates (in Å) for the optimized (loose threshold, see Table 4 in the paper) lowest-lying $\pi\pi^*$ state of thymine obtained at the CASPT2(10,8)/6-31G* level of theory.

C1	−1.652767	0.047180	1.499857
C2	0.242671	−1.554130	1.500180
C3	1.159597	−0.452737	1.499387
C4	2.631292	−0.694825	1.500281
C5	0.707750	0.894854	1.499216
N1	−0.744414	1.045722	1.499015
N2	−1.100805	−1.248089	1.499917
O1	1.396121	1.953632	1.499623
O2	−2.896630	0.150222	1.500369
H1	0.517091	−2.601715	1.500679
H2	−1.091307	2.000352	1.499570
H3	−1.811705	−1.980006	1.500158
H4	2.864085	−1.764706	1.493166
H5	3.093412	−0.238552	2.382508
H6	3.095843	−0.225814	0.626185

Table S34. Cartesian coordinates (in Å) for the optimized (loose threshold, see Table 4 in the paper) lowest-lying $\pi\pi^*$ state of thymine obtained at the MS-CASPT2(10,8)/6-31G* level of theory.

C1	−1.650463	0.046630	1.499902
C2	0.244662	−1.554598	1.500019
C3	1.158035	−0.454053	1.499348
C4	2.629756	−0.695157	1.500418
C5	0.708276	0.894599	1.498968
N1	−0.745154	1.046788	1.498837
N2	−1.099991	−1.248544	1.500187
O1	1.396976	1.951344	1.499294
O2	−2.895417	0.149899	1.500477
H1	0.521132	−2.601591	1.500074
H2	−1.090875	2.001721	1.499240
H3	−1.812362	−1.978662	1.500908
H4	2.862586	−1.764770	1.492632
H5	3.090154	−0.238111	2.382923
H6	3.092919	−0.224108	0.626885

Table S35. Cartesian coordinates (in Å) for the optimized (tight threshold, see Table 4 in the paper) lowest-lying $\pi\pi^*$ state of thymine obtained at the CASPT2(10,8)/6-31G* level of theory.

C1	−1.282418	0.050136	1.923989
C2	0.199024	−1.518140	0.917579
C3	1.228071	−0.476481	0.834781
C4	2.328880	−0.666679	1.877804
C5	0.742521	0.854932	0.745733
N1	−0.699640	0.913454	1.040549
N2	−0.719052	−1.306282	1.854773
O1	1.341649	1.932019	0.678673
O2	−2.277302	0.214899	2.608943
H1	0.117859	−2.419247	0.302368
H2	−1.061664	1.862771	1.101724
H3	−1.371017	−2.016737	2.198628
H4	3.089488	−1.377609	1.536985
H5	1.961974	−1.020320	2.853952
H6	2.811859	0.304672	2.023631

Table S36. Cartesian coordinates (in Å) for the optimized (tight threshold, see Table 4 in the paper) lowest-lying $\pi\pi^*$ state of thymine obtained at the MS-CASPT2(10,8)/6-31G* level of theory.

C1	−1.617882	0.063080	1.400384
C2	0.248308	−1.543150	1.607725
C3	1.164857	−0.447372	1.635529
C4	2.611272	−0.698383	1.379675
C5	0.696358	0.891133	1.767007
N1	−0.753446	0.991935	1.868398
N2	−1.044357	−1.224885	1.266632
O1	1.335805	1.965564	1.742487
O2	−2.827508	0.191343	1.150553
H1	0.464481	−2.577319	1.865468
H2	−1.100061	1.947459	1.906542
H3	−1.721594	−1.929885	0.967488
H4	2.999409	−1.477025	2.049091
H5	3.167512	0.226071	1.541658
H6	2.787079	−1.047178	0.351476

Table S37. Cartesian coordinates (in Å) for the final structure in the minimum energy path (r = 0.09) along lowest-lying $\pi\pi^*$ state of thymine obtained at the CASPT2(10,8)/6-31G* level of theory.

C1	−1.6133680	0.0713793	1.3925476
C2	0.2214594	−1.5633231	1.6446532
C3	1.1716253	−0.4677741	1.6758839
C4	2.6139270	−0.6928633	1.3653135
C5	0.7010819	0.8728837	1.7872755
N1	−0.7514791	0.9874541	1.8996292
N2	−1.0418395	−1.2288367	1.2529312
O1	1.3408750	1.9490856	1.7282029
O2	−2.8113867	0.2072203	1.1213474
H1	0.4203948	−2.5999364	1.9065117
H2	−1.0889484	1.9469542	1.9201688
H3	−1.7214160	−1.9255803	0.9375575
H4	3.0469500	−1.4637668	2.0167767
H5	3.1433849	0.2499456	1.5250435
H6	2.7789719	−1.0114548	0.3262688

Table S38. Cartesian coordinates (in Å) for the final structure in the minimum energy path (r = 0.18) along lowest-lying $\pi\pi^*$ state of thymine obtained at the CASPT2(10,8)/6-31G* level of theory.

C1	−1.246109	0.016529	1.048383
C2	0.210162	−1.511522	2.135362
C3	1.247453	−0.480909	2.209809
C4	2.287302	−0.642618	1.093567
C5	0.742255	0.854738	2.274564
N1	−0.684412	0.909815	1.920750
N2	−0.700043	−1.338381	1.182310
O1	1.328622	1.933128	2.374044
O2	−2.216814	0.170525	0.327529
H1	0.123353	−2.398556	2.770352
H2	−1.046295	1.856321	1.822161
H3	−1.354662	−2.058099	0.864775
H4	2.927573	−1.514240	1.261571
H5	2.917161	0.250808	1.140144
H6	1.874687	−0.716150	0.074790

Table S39. Cartesian coordinates (in Å) for the final structure in the minimum energy path (r = 0.18) along lowest-lying $\pi\pi^*$ state of thymine obtained at the MS-CASPT2(10,8)/6-31G* level of theory.

C1	−1.601155	0.070384	1.358302
C2	0.227149	−1.549654	1.675672
C3	1.170099	−0.448667	1.689648
C4	2.600369	−0.696749	1.343284
C5	0.701370	0.882275	1.824088
N1	−0.751828	0.983259	1.882530
N2	−1.025744	−1.231282	1.246342
O1	1.338981	1.958411	1.811815
O2	−2.792781	0.208592	1.060314
H1	0.423408	−2.578690	1.969350
H2	−1.098538	1.938500	1.925822
H3	−1.702786	−1.935225	0.941057
H4	3.042403	−1.457534	2.001654
H5	3.149975	0.240071	1.459997
H6	2.729309	−1.052305	0.310238

Table S40. Cartesian coordinates (in Å) for the final structure in the minimum energy path (r = 0.30) along lowest-lying $\pi\pi^*$ state of thymine obtained at the CASPT2(10,8)/6-31G* level of theory.

C1	−1.234657	0.011329	1.042828
C2	0.215287	−1.511330	2.152454
C3	1.245859	−0.478249	2.217011
C4	2.270965	−0.635761	1.083602
C5	0.743752	0.856082	2.281950
N1	−0.681093	0.905705	1.915178
N2	−0.696213	−1.345720	1.201589
O1	1.328106	1.935554	2.380528
O2	−2.192866	0.162755	0.304790
H1	0.135011	−2.396322	2.791040
H2	−1.041171	1.852245	1.808529
H3	−1.348107	−2.067859	0.884079
H4	2.874725	−1.540460	1.205289
H5	2.940286	0.225775	1.166027
H6	1.850350	−0.642357	0.065216

Table S41. Cartesian coordinates (in Å) for the final structure in the minimum energy path (r = 0.30) along lowest-lying $\pi\pi^*$ state of thymine obtained at the MS-CASPT2(10,8)/6-31G* level of theory.

C1	−1.130686	−0.030641	0.969779
C2	0.225765	−1.524846	2.225329
C3	1.279067	−0.519402	2.278202
C4	2.179761	−0.584338	1.029370
C5	0.746960	0.831377	2.384450
N1	−0.596665	0.913500	1.810198
N2	−0.677468	−1.384896	1.254399
O1	1.286367	1.891612	2.678348
O2	−2.029014	0.122789	0.163122
H1	0.102698	−2.380114	2.896738
H2	−0.941636	1.858633	1.655376
H3	−1.348707	−2.102889	0.970568
H4	2.732872	−1.528113	0.994253
H5	2.907512	0.225072	1.132473
H6	1.673407	−0.456357	0.057506

Table S42. Cartesian coordinates (in Å) for the optimized $n\sigma\pi^*$ minimum of thymine obtained at the CASSCF(14,10)/6-31G* level of theory.

N	−0.74931716	1.05623131	1.59450530
N	−1.13505381	−1.22780031	1.57647533
C	−1.67360893	0.02816228	1.53624698
C	0.23612619	−1.51175143	1.54377333
C	1.15049220	−0.45796639	1.47821733
C	2.64030916	−0.69531520	1.49265385
C	0.61979398	0.81409925	1.42814701
O	1.37228190	1.94651904	1.58649365
O	−2.85290498	0.23695128	1.46700909
H	0.50884647	−2.54359187	1.48713498
H	−1.11308100	1.93627727	1.30068190
H	−1.79863621	−1.96666629	1.53797969
H	2.85736554	−1.74938270	1.37013397
H	3.08047343	−0.37153343	2.43046101
H	3.13577284	−0.15892423	0.69104539

Table S43. Cartesian coordinates (in Å) for the optimized nσπ* minimum of thymine obtained at the CASPT2(14,10)/6-31G* level of theory.

N	−0.74348063	1.05228220	1.67019066
N	−1.13594506	−1.23747205	1.57070143
C	−1.67955338	0.02505451	1.50646750
C	0.23804621	−1.52531305	1.49661253
C	1.15287186	−0.46756974	1.51858375
C	2.63920410	−0.69059751	1.47708559
C	0.63176254	0.80789572	1.52176904
O	1.37318288	1.95266584	1.58035346
O	−2.87812755	0.25147183	1.37566850
H	0.51716130	−2.56832181	1.55608430
H	−1.10229216	1.96760658	1.41445536
H	−1.81430750	−1.97708224	1.43061980
H	2.86401364	−1.75908366	1.51650871
H	3.13584077	−0.20915611	2.32556934
H	3.08048261	−0.28707194	0.56028884

Table S44. Cartesian coordinates (in Å) for the optimized nσπ* minimum of thymine obtained at the MS-CASPT2(14,10)/6-31G* level of theory.

N	−0.74323645	1.05292242	1.66367002
N	−1.13569326	−1.23724846	1.57624029
C	−1.67974991	0.02499609	1.50781750
C	0.23807393	−1.52532398	1.49888468
C	1.15285386	−0.46750407	1.51584568
C	2.63925743	−0.69062486	1.47831500
C	0.63180716	0.80803081	1.51520969
O	1.37362235	1.95275502	1.56893346
O	−2.87866425	0.25077221	1.37967727
H	0.51757209	−2.56798622	1.56253464
H	−1.10275576	1.96724955	1.40557578
H	−1.81451587	−1.97727030	1.44052287
H	2.86278281	−1.76008946	1.48314510
H	3.13025671	−0.23829438	2.34593247
H	3.08724877	−0.25707578	0.57865435

Table S45. Cartesian coordinates (in Å) for the ππ*/nσπ* conical intersection of thymine obtained at the CASSCF(14,10)/6-31G* level of theory.

N	−0.76300530	1.00348908	1.72194730
N	−0.84947975	−1.29736725	1.45774290
C	−1.45058364	−0.03691747	1.19116607
C	0.19368440	−1.36764874	2.25821089
C	1.22547886	−0.27029355	2.04153925
C	2.43453448	−0.72976706	1.25689175
C	0.67436547	0.98313379	1.81079618
O	1.22469868	2.05789602	1.56102236
O	−2.50403883	−0.00280632	0.61635909
H	0.41800910	−2.31430593	2.71067701
H	−1.16598309	1.90081759	1.54963517
H	−1.41982803	−2.08290409	1.20443364
H	2.17540884	−1.35195683	0.39617576
H	3.13288867	−1.30071512	1.86387556
H	2.95270976	0.14465447	0.88048585

Table S46. Cartesian coordinates (in Å) for the ππ*/nσπ* conical intersection of thymine obtained at the CASPT2(14,10)/6-31G* level of theory.

N	−0.73837247	1.05533513	1.53417213
N	−1.11799576	−1.24375427	1.48549142
C	−1.64585529	0.04279301	1.49170990
C	0.23823753	−1.56184491	1.51146267
C	1.14337363	−0.45768916	1.50415851
C	2.61864985	−0.69930284	1.49847429
C	0.69311071	0.87949570	1.49101550
O	1.40434649	1.93612056	1.48732863
O	−2.89390277	0.17824270	1.49652569
H	0.50394725	−2.60592832	1.58365436
H	−1.09115910	2.00441076	1.50705333
H	−1.82830830	−1.97206457	1.43881608
H	2.85272662	−1.76613339	1.47377362
H	3.07245556	−0.24780306	2.38569745
H	3.06760567	−0.20656874	0.63162521

Table S47. Cartesian coordinates (in Å) for the ππ*/nOπ* conical intersection of thymine obtained at the MS-CASPT2(14,10)/6-31G* level of theory.

N	−0.73837247	1.05533513	1.53417213
N	−1.11799576	−1.24375427	1.48549142
C	−1.64585529	0.04279301	1.49170990
C	0.23823753	−1.56184491	1.51146267
C	1.14337363	−0.45768916	1.50415851
C	2.61864985	−0.69930284	1.49847429
C	0.69311071	0.87949570	1.49101550
O	1.40434649	1.93612056	1.48732863
O	−2.89390277	0.17824270	1.49652569
H	0.50394725	−2.60592832	1.58365436
H	−1.09115910	2.00441076	1.50705333
H	−1.82830830	−1.97206457	1.43881608
H	2.85272662	−1.76613339	1.47377362
H	3.07245556	−0.24780306	2.38569745
H	3.06760567	−0.20656874	0.63162521

Table S48. Geometrical parameters describing the different structures obtained with the CASSCF method and different geometry optimization and MEP procedures and two active spaces. Distances are given in Å and angles in degrees.

Type of Calc.	Basis Set	RMS Diff. [1]	Dihedral/HC6C5C7	Dist C6-C5	Dist C5-C7
Opt 10in8	6-31G	0.03510808	0.2212	1.4113	1.5029
	6-31G*	0.00370932	0.238	1.5076	1.499
	ANO-S 321/21	0.00476646	0.1126	1.503	1.5002
	ANO-L 321/21	0.00441282	0.0835	1.5028	1.4989
	ANO-L 432/21	0	0.1125	1.4976	1.4957
Opt Tight [2] 10in8	6-31G	0.04963546	1.0194	1.4113	1.5029
	6-31G*	0.00339285	117.0935	1.4517	1.5527
	ANO-S 321/21	0.00485704	117.9988	1.4537	1.5561
	ANO-L 321/21	0.00503257	−117.3786	1.4524	1.5556
	ANO-L 432/21	0	116.9454	1.4478	1.5594
Opt 10in11	6-31G	0.04985762	0.3373	1.4096	1.5026
	6-31G*	0.00259809	117.978	1.4511	1.5545
	ANO-S 321/21	0.00452152	−0.8654	1.4859	1.5005
	ANO-L 321/21	0.00422125	117.7202	1.4524	1.5547
	ANO-L 432/21	0	118.1073	1.4506	1.5544
Opt Tight [2] 10in11	6-31G	0.05091396	0.0309	1.4095	1.5026
	6-31G*	0.00815668	116.6511	1.4516	1.5544
	ANO-S 321/21	0.00930307	117.431	1.4555	1.5559
	ANO-L 321/21	0.01100682	117.2557	1.4373	1.5524
	ANO-L 432/21	0	121.3845	1.4419	1.5566
MEP 10in8	6-31G	0.05088403	−2.108	1.4114	1.5028
	6-31G*	0.00652522	115.806	1.4518	1.5524
	ANO-S 321/21	0.00627211	−116.8655	1.4542	1.5559
	ANO-L 321/21	0.00616924	116.8377	1.4526	1.5555
	ANO-L 432/21	0	−116.9454	1.4409	1.5516
MEP 10in11	6-31G	0.04574755	−0.6271	1.41	1.5026
	6-31G*	0.00755868	117.2581	1.4516	1.5539
	ANO-S 321/21	0.00832966	118.6335	1.4547	1.5493
	ANO-L 321/21	0.00908163	118.4396	1.4552	1.5552
	ANO-L 432/21	0	118.1398	1.4508	1.5545

[1] Root Mean Square (RMS) differences for all bonds with respect to the $^1(\pi\pi^*/S_0)_{CI}$ and $^1(\pi\pi^*)_{min}$ geometries obtained at the CASSCF/ANO-L 432/21 level of theory; [2] Convergence thresholds of 10^{-8} and 10^{-6} for the energy change and the norm of the gradient, respectively.

Table S49. Geometrical parameters describing the differences between the pairs of CASSCF(10,8) and CASSCF(10,11) structures optimized with distinct basis sets (6-31G, 6-31G*, ANO-S 321/21, ANO-L 321/21, and ANO-L 432/21) and geometry optimization techniques (Opt, Opt Tight, and MEP).

Type of Calc.	Basis Set	RMS Diff.	ΔDihedral/HC6C5C7	ΔDist C6-C5	ΔDist C5-C7
Opt	6-31G	0.00213599	0.1161	−0.0017	−0.0003
	6-31G*	0.02665290	117.74	−0.0565	0.0555
	ANO-S 321/21	0.02458788	−0.978	−0.0171	0.0003
	ANO-L 321/21	0.02482216	117.6367	−0.0504	0.0558
	ANO-L 432/21	0.02520079	117.9948	−0.047	0.0587
Opt Tight [1]	6-31G	0.00211656	0.3064	0.0004	0
	6-31G*	0.00162878	1.3269	−0.0005	0.0004
	ANO-S 321/21	0.00136347	118.2964	0.0304	−0.0554
	ANO-L 321/21	0.00509343	0.4645	0.0151	0.0023
	ANO-L 432/21	0.00538192	3.2772	0.0087	−0.0022
MEP	6-31G	0.00224780	1.4809	−0.0014	−0.0002
	6-31G*	0.00258715	1.4521	−0.0002	0.0015
	ANO-S 321/21	0.00404981	1.768	0.0005	−0.0066
	ANO-L 321/21	0.00158425	1.6019	0.0026	−0.0003
	ANO-L 432/21	0.00453224	1.1944	0.0099	0.0029

[1] Convergence thresholds of 10^{-8} and 10^{-6} for the energy change and the norm of the gradient, respectively.

Table S50. Absolute S_0 and S_1 energies at the S_0 equilibrium structure and at the $\pi\pi^*$ geometries obtained with distinct level of theory. The representative 6-31G* basis set is used in all cases.

	S_0(CASSCF)	S_1(CASSCF)	S_0(CASPT2)	S_1(CASPT2)	S_0(MSPT2)[1]	S_1(MSPT2)[1]
FC S_0 min	−451.5732686	−451.3295897	−452.8056033	−452.6223005	−452.8057800	−452.6221292
CASSCF opt 10in8 loose	−451.5415234	−451.3555102	−452.7781229	−452.6292959	−452.7791006	−452.6283182
CASSCF opt 10in8 tight	−451.4010233	−451.3966625	−452.6479312	−452.6408158	−452.6511648	−452.6375822
CASSCF MEP 10in8	−451.4030672	−451.3967661	−452.6493238	−452.6409617	−452.6537733	−452.6365123
CASSCF opt 10in11 loose	−451.4440643	−451.4438087	−452.6438164	−452.6384756	−452.6488901	−452.6334019
CASSCF opt 10in11 tight	−451.4473687	−451.4432698	−452.6460971	−452.6383481	−452.6515173	−452.6329279
CASSCF MEP 10in11	−451.4452026	−451.4342683	−452.6466749	−452.6352455	−452.647564	−452.6343563
CASPT2 opt 10in8 loose	−451.5470816	−451.3476991	−452.7929359	−452.6380433	−452.7934495	−452.6375297
CASPT2 opt 10in8 tight	−451.4173599	−451.3858558	−452.6704426	−452.647014	−452.6771046	−452.640352
MSPT2[1] opt 10in8 loose	−451.5472208	−451.347692	−452.7931193	−452.6380795	−452.7935923	−452.6376065
MSPT2[1] opt 10in8 tight	−451.5288935	−451.3532834	−452.7747857	−452.6398807	−452.7756751	−452.6389913
CASPT2 MEP 10in8 r = 0.09	−451.5219043	−451.3557397	−452.7684716	−452.6398827	−452.7695481	−452.6388062
CASPT2 MEP 10in8 r = 0.18	−451.4130668	−451.387413	−452.666077	−452.6466238	−452.6729616	−452.6397393
CASPT2 MEP 10in8 r = 0.30	−451.3920284	−451.3732461	−452.6669273	−452.6520645	−452.6712552	−452.6477366
MSPT2[1] MEP 10in8 r = 0.18	−451.5184721	−451.3580508	−452.7651546	−452.6399908	−452.7662975	−452.6388478
MSPT2[1] MEP 10in8 r = 0.30	−451.405041	−451.3881352	−452.658783	−452.6427319	−452.6588209	−452.6426941

[1] MSPT2 stands for MS-CASPT2.

Table S51. Absolute S_0, S_1, and S_2 energies at the S_0 equilibrium structure and at the 14in10 $n\pi^*$ geometries obtained with distinct levels of theory. The representative 6-31G* basis set is used in all cases.

	S_0(CASSCF)	S_1(CASSCF)	S_2(CASSCF)	S_0(CASPT2)	S_1(CASPT2)	S_2(CASPT2)	S_0(MSPT2)[1]	S_1(MSPT2)[1]	S_2(MSPT2)[1]
FC S_0 min	−451.5686988	−451.3745857	−451.3169647	−452.8023498	−452.6252660	−452.6227746	−452.80415131	−452.6253023	−452.6224275
CASSCF $n_O\pi^*$ min	−451.5215223	−451.4172627	−451.3358571	−452.763113	−452.6478682	−452.598085	−452.770753	−452.6485317	−452.6240226
CASPT2 $n_O\pi^*$ min	−451.5219152	−451.413579	−451.3371539	−452.7697119	−452.6505823	−452.6062331	−452.7761942	−452.651241	−452.6249708
MS-PT2 $n_O\pi^*$ min	−451.5219738	−451.4135866	−451.3371565	−452.7697474	−452.6505778	−452.6062021	−452.7762021	−452.6512356	−452.6248883
CASSCF $\pi\pi^*$/$n_O\pi^*$ CI	−451.4598747	−451.3561518	−451.3524422	−452.7049745	−452.6412235	−452.5938754	−452.717764	−452.6332765	−452.5923073
CASPT2 $\pi\pi^*$/$n_O\pi^*$ CI	−451.5403529	−451.3908686	−451.3339469	−452.7869879	−452.6346595	−452.6349052	−452.790833	−452.636729	−452.6359589
MSPT2[1] $\pi\pi^*$/$n_O\pi^*$ CI	−451.5403529	−451.3908686	−451.3339469	−452.7869879	−452.6346595	−452.6349052	−452.790833	−452.636729	−452.6359589

[1] MSPT2 stands for MS-CASPT2.

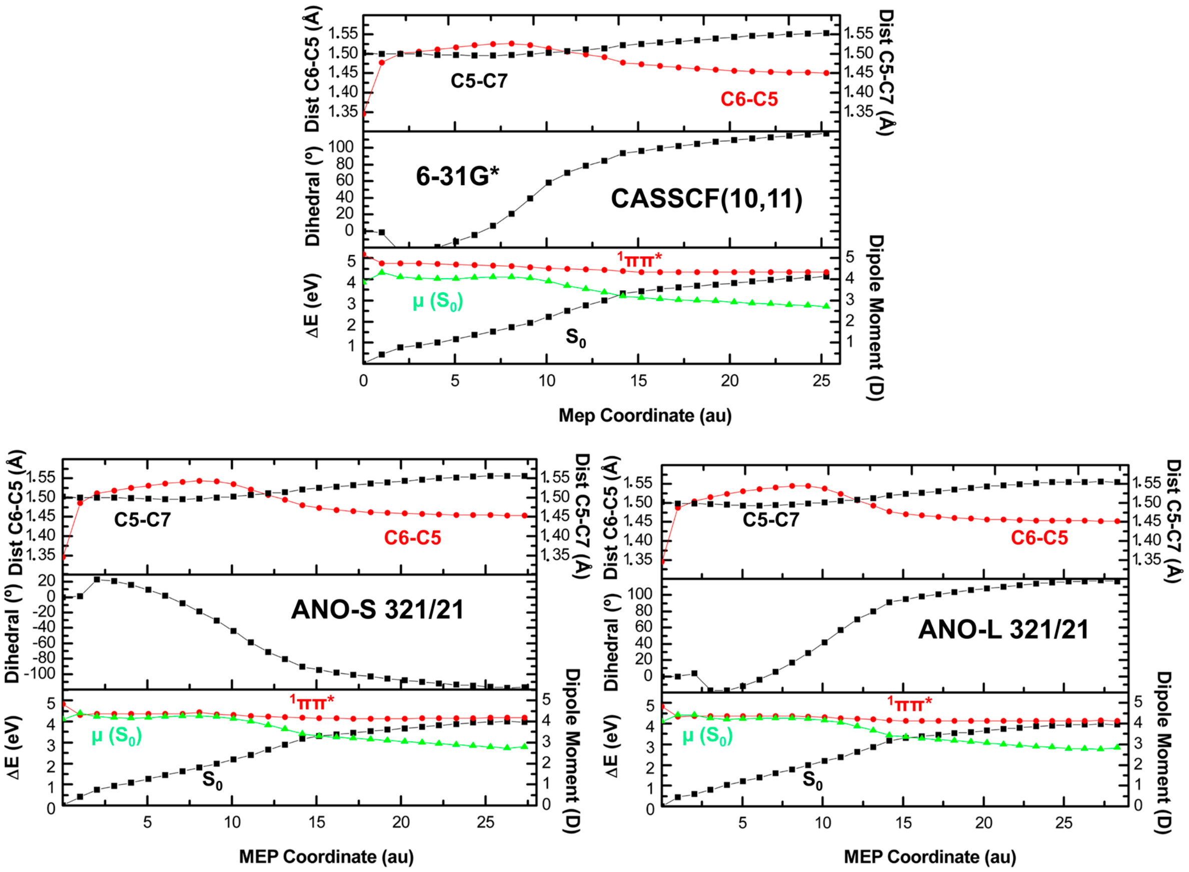

Figure S1. Comparison of the MEPs of the $^1(\pi\pi^*)$ state of thymine computed at the CASSCF(10,11)/6-31G*, CASSCF(10,8)/ ANO-S 312/21, and CASSCF(10,8)/ ANO-L 321/21 levels of theory.

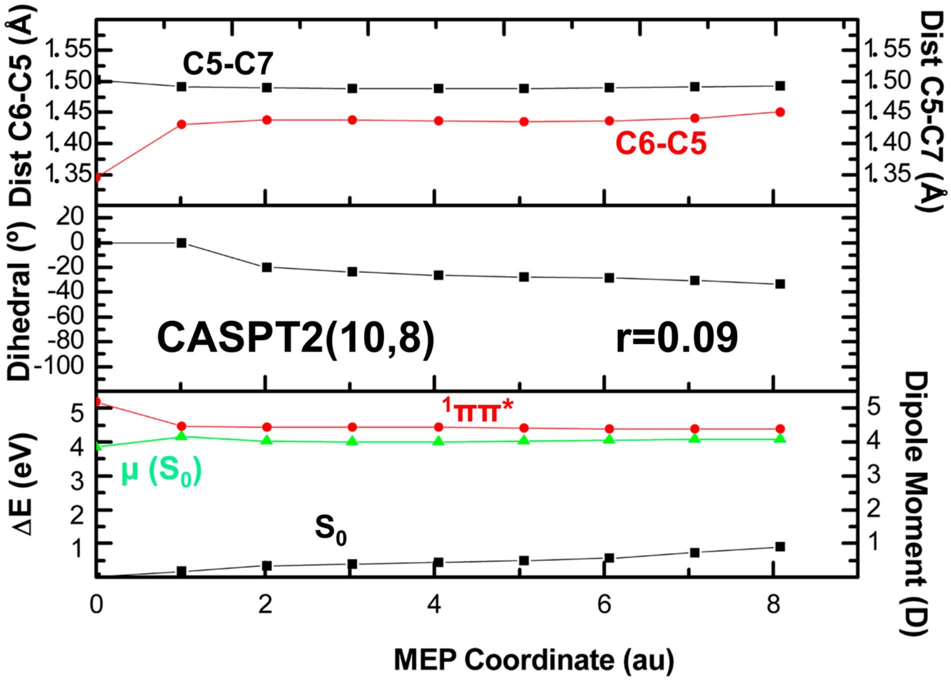

Figure S2. MEP of the $^1(\pi\pi^*)$ state of thymine computed at the CASPT2//CASPT2 level of theory making use of a hypersphere of 0.09 a.u.
